# Inhibited Personality Temperaments Translated Through Enhanced Avoidance and Associative Learning Increase Vulnerability for PTSD

**DOI:** 10.3389/fpsyg.2019.00496

**Published:** 2019-03-22

**Authors:** Michael Todd Allen, Catherine E. Myers, Kevin D. Beck, Kevin C. H. Pang, Richard J. Servatius

**Affiliations:** ^1^ School of Psychological Sciences, University of Northern Colorado, Greeley, CO, United States; ^2^ Rutgers Biomedical Health Sciences, Stress and Motivated Behavior Institute, Rutgers University, Newark, NJ, United States; ^3^ Central New York Research Corporation, Syracuse, NY, United States; ^4^ Department of Veterans Affairs, VA New Jersey Health Care System, East Orange, NJ, United States; ^5^ Department of Pharmacology, Physiology and Neuroscience, Rutgers University-New Jersey Medical School, Newark, NJ, United States; ^6^ Department of Veterans Affairs, Syracuse Veterans Affairs Medical Center, Syracuse, NY, United States; ^7^ Department of Psychiatry, State University of New York Upstate Medical University, Syracuse, NY, United States

**Keywords:** PTSD, personality temperaments, associative learning, rat model, avoidance learning, eyeblink conditioning

## Abstract

Although many individuals who experience a trauma go on to develop post-traumatic stress disorder (PTSD), the rate of PTSD following trauma is only about 15–24%. There must be some pre-existing conditions that impart increased vulnerability to some individuals and not others. Diathesis models of PTSD theorize that pre-existing vulnerabilities interact with traumatic experiences to produce psychopathology. Recent work has indicated that personality factors such as behavioral inhibition (BI), harm avoidance (HA), and distressed (Type D) personality are vulnerability factors for the development of PTSD and anxiety disorders. These personality temperaments produce enhanced acquisition or maintenance of associations, especially avoidance, which is a criterion symptom of PTSD. In this review, we highlight the evidence for a relationship between these personality types and enhanced avoidance and associative learning, which may increase risk for the development of PTSD. First, we provide the evidence confirming a relationship among BI, HA, distressed (Type D) personality, and PTSD. Second, we present recent findings that BI is associated with enhanced avoidance learning in both humans and animal models. Third, we will review evidence that BI is also associated with enhanced eyeblink conditioning in both humans and animal models. Overall, data from both humans and animals suggest that these personality traits promote enhanced avoidance and associative learning, as well as slowing of extinction in some training protocols, which all support the learning diathesis model. These findings of enhanced learning in vulnerable individuals can be used to develop objective behavioral measures to pre-identify individuals who are more at risk for development of PTSD following traumatic events, allowing for early (possibly preventative) intervention, as well as suggesting possible therapies for PTSD targeted on remediating avoidance or associative learning. Future work should explore the neural substrates of enhanced avoidance and associative learning for behaviorally inhibited individuals in both the animal model and human participants.

Post-traumatic stress disorder (PTSD) is a devastating mental illness that can develop following exposure to a traumatic event. However, it is unclear why only a percentage (15–24%) of individuals who experience trauma go on to develop PTSD (Breslau et al., 1998). For example, a study of New York City residents living close to the site of the 9/11 World Trade Center terrorist attacks reported that only about 12.6% had probable PTSD, based on self-reported symptoms (DiGrande et al., 2008). Another recent study of US military personnel deployed to operations in Iraq or Afghanistan reported a PTSD rate of about 6% (Jacobson et al., 2015). Thus, individuals who experience highly traumatic events differ in how they process and recover from that event.

In addition, even among individuals who develop PTSD, there is wide variability in symptom presentation. In the fifth edition of the *Diagnostic and Statistical Manual* (DSM-5; American Psychiatric Association, 2013), PTSD is now categorized as a trauma- and stressor-related disorder rather than an anxiety disorder. In the DSM-5, PTSD symptoms are grouped into five criteria, which include exposure to a stressor, intrusion symptoms such as re-experiencing, avoidance of trauma-related stimuli, negative alterations in cognitions and mood, and alterations in arousal and reactivity. However, individuals vary in the degree to which they meet these criteria. In fact, it has been calculated that over 636,000 possible presentations could satisfy DSM-5 definitional criteria for PTSD (Galatzer-Levy and Bryant, 2013). The individual differences in symptom severity following comparable traumatic events (Pitman et al., 1987; Orr et al., 1993; Shalev et al., 1993) suggest that pre-existing vulnerabilities may modulate the degree to which an individual develops PTSD or is resilient in the face of trauma. It is currently unclear which factors determine how an individual reacts to a traumatic event to either develop PTSD or display resilience.

## Diathesis Models

Diathesis models of mental illness explain psychiatric disorders such as PTSD as coming about through dynamic interactions of pre-existing vulnerabilities including genes, epigenetics, and personality with exposure to trauma. While it is accepted that PTSD results from an exposure to a traumatic stressor (e.g., North and Smith, 1990; Davidson, 2000; Seng et al., 2009), there is still much debate and controversy surrounding the exact nature of the exposure to a traumatic event as details of Criterion A were altered in the most recent revision of the DSM (for a review, see Pai et al., 2017). Regardless of the definition, trauma has a necessary but insufficient role in the development of PTSD in which several other vulnerability factors have been identified, including genes (Binder et al., 2008; Amstadter et al., 2009), neural abnormalities (Pitman et al., 2006; Liberzon and Sripada, 2008), and personality traits (Engelhard et al., 2006; Gil and Caspi, 2006; Qouta et al., 2007). Recent work in rodents and humans has focused on personality traits that produce behavioral and learning tendencies, which lead to the development of PTSD.

One behavioral tendency, which has received much attention, is avoidance. Although disorders such as generalized anxiety disorder (GAD), obsessive-compulsive disorder (OCD), panic disorder, social anxiety disorder (SAD), social phobia, and PTSD have distinguishing symptoms, excessive or maladaptive avoidance is a common symptom in all these disorders. To satisfy *DSM-5* Criterion C for PTSD, an individual must persistently avoid either external reminders of trauma (i.e., behavioral avoidance) or trauma-related thoughts or feelings (i.e., cognitive avoidance), or both. Avoidance of traumatic or stressful situations is usually adaptive in that it protects from possible harm. However, avoidance can become maladaptive, limit social and non-social interactions, and disrupt normal life functioning as in the case of PTSD. Moreover, avoidance contributes to the chronicity of disorder as it prevents the individual from learning that certain situations or stimuli are not or no longer dangerous.

Avoidant tendencies may be particularly important in differentiating individuals likely to develop PTSD following exposure to a traumatic event from those who display resilience in the face of trauma. Avoidance or escape behaviors have been identified as a significant predictor of PTSD (Charlton and Thompson, 1996; Marmar, 1996; Chang et al., 2003; Gil and Caspi, 2006) as well as distinguishing between those at risk and not at risk for development of PTSD or anxiety disorders (North et al., 1999; Barlow, 2002; Karamustafalioglu et al., 2006; Marshall et al., 2006; O’Donnell et al., 2007). Specifically, avoidance only occurs in less than half of trauma-exposed individuals (Maes et al., 1998; Breslau et al., 1999), but individuals who report avoidance symptoms following a trauma have an increased likelihood of developing PTSD (North et al., 1999). In addition, the consistency and intensity of avoidance symptoms (Solomon et al., 2009) may be related to the persistence (Maes et al., 1998) and full expression of PTSD (North et al., 2004; Karamustafalioglu et al., 2006; Kashdan et al., 2006; O’Donnell et al., 2007). Thus, the acquisition, expression, and retention of avoidance may represent an endophenotype (Gould and Gottesman, 2006) for PTSD and be the final common pathway to PTSD. The integral role of avoidance in PTSD has also led to renewed interest in avoidance learning as a valuable experimental paradigm in animal and human models of PTSD for the study of neural and behavioral abnormalities in PTSD (e.g., Lovibond, 2006; Servatius, 2016; LeDoux et al., 2017; Pittig et al., 2018).

Besides avoidance, diagnostic symptom criteria for PTSD involve increased arousal and reactivity to stimuli and events associated with the traumatic event (Criterion E in the *DSM-5*). One expression of this altered arousal and reactivity is hypervigilance. Hypervigilance can be expressed in several ways. An individual may express general hypervigilance by over-attending to possible aversive stimuli by searching for possible threats (Mathews, 1994; McNally, 1996). Hypervigilance may ultimately result in avoidance if an individual over-attends to an aversive stimulus and then subsequently avoids that same stimuli (Bögels and Mansell, 2004). In addition, hypervigilance may also produce enhanced associative learning with aversive stimuli. Thus, hypervigilance may underlie the development of PTSD through avoidance learning as well as associative learning.

In the current review, we will present recent findings supporting a learning diathesis model in which avoidance-related personality factors related to PTSD produce enhanced avoidance and associative learning. These convergent findings come from rodents and humans including non-clinical and clinical samples. First, we will review several personality temperaments that have been suggested to be risk factors for the development of PTSD through avoidance and hypervigilance. Second, we will present findings of enhanced avoidance learning in humans and an animal model of PTSD vulnerability. Third, we will detail findings of enhanced acquisition and slowed extinction of associative learning in behaviorally inhibited as well as PTSD individuals. The neural substrates of avoidance and associative learning tasks will be discussed in the context of understanding how these learning mechanisms may produce and maintain PTSD.

## PTSD Vulnerability Factors

Recent work has identified several avoidant or inhibited personality temperaments that increase risk for the development of PTSD. In addition, these inhibited temperaments have been linked to enhanced learning, which supports a learning diathesis model of PTSD.

One personality trait linked to PTSD is trait anxiety, which is defined as a relatively stable bias to perceive events as threatening. Trait anxiety can be measured by the State Trait Anxiety Inventory (STAI; Spielberger, 1983). Individuals diagnosed with PTSD exhibit higher levels of trait anxiety than non-PTSD individuals (e.g., Orsillo et al., 1996; Casada and Roache, 2005; Casada and Roache, 2006). Highly trait anxious individuals display hypervigilance (Eysenck and Calvo, 1992), which we have discussed above as a mechanism for PTSD development. In addition, higher rates of PTSD symptoms were reported 2–5 years following surgery for those individuals with high levels of trait anxiety at the time of the operation (Ristvedt and Trinkaus, 2009). This finding suggests that trait anxiety may be a pre-existing vulnerability factor, rather than a trait that emerges solely following trauma exposure and/or development of PTSD. In fact, some researchers have suggested that prospective trait anxiety is a better predictor of PTSD symptoms than the nature of the traumatic event (Lonigan et al., 1994; Phipps et al., 2009).

Another personality temperament that has been linked to PTSD is behavioral inhibition (BI), which is defined as a temperamental tendency to avoid novel individuals and situations (Kagan et al., 1987; Morgan, 2006). BI can be measured with the Adult and Retrospective Measures of Behavioral Inhibition (AMBI/RMBI; Gladstone et al., 2005; Gladstone and Parker, 2005). BI as measured by the AMBI is positively related to trait but not state anxiety (Caulfield et al., 2013). BI expressed during childhood increases the risk for an individual to develop PTSD (North et al., 1999; Fincham et al., 2008; Kashdan et al., 2009) as well as anxiety disorders (Hirshfeld et al., 1992; Biederman et al., 1993; Svihra and Katzman, 2004; Pérez-Edgar et al., 2010) in adulthood. Several aspects of BI are related to PTSD symptoms. For example, BI involves increased sensitivity to novel, threatening, and uncertain situations and stimuli (Hirshfeld et al., 1992; Schwartz et al., 2003a,b; Smoller et al., 2005) as well as increased physiological reactivity to stressful stimuli (Keltikangas-Jarvinen et al., 1999; Kalin et al., 2000; Kalin and Shelton, 2003; Schwartz et al., 2003a,b; Smoller et al., 2005; Tyrka et al., 2006, 2008; Perez-Edgar et al., 2007).

Recent work has demonstrated a relationship between BI and PTSD in a variety of samples. AMBI, but not RMBI, scores were associated with scores on the PTSD checklist (PCL-M) administered to Veterans (Myers et al., 2012a,b). This relationship was the strongest for avoidance symptoms (i.e., PTSD symptom cluster C as defined in the DSM-IV). BI was also related to PTSD symptom severity in a sample of active duty Coast Guard personnel (Servatius et al., 2017). In this study, PTSD was measured with the PTSD checklist for both military and non-military experiences. Similarly, in a sample of active duty Coast Guard personnel, individuals meeting symptom criteria for PTSD had higher rates of BI than individuals not meeting symptom criteria (76 versus 30%, respectively). Therefore, there is a high concordance between PTSD and BI in military samples (Handy et al., 2017).

Other personality factors found to be strongly related to BI are harm avoidance or HA (Allen et al., 2017) and distressed (Type D) personality (Allen et al., 2018). HA is defined as a tendency to avoid punishing, novel, and non-rewarding situations (Nixon and Parsons, 1989) and can be measured with a subscale of the Tridimensional Personality Questionnaire (TPQ; Cloninger et al., 1991, 1993). High levels of HA are a risk factor for PTSD (Gil, 2005), while low levels are associated with resilience (Simeon et al., 2007).

Type D personality is measured with the Type D scale (DS-14, Denollet, 2005) and involves high levels of both social inhibition (SI) and negative affectivity (NA). High NA results in negative emotions, sadness, and a gloomy outlook on life, while high SI would result in a tendency to not share these negative emotions with others for fear of their reactions. Type D (including both the SI and NA subscales) was significantly positively correlated with current BI in a non-clinical sample (Allen et al., 2018) and is also related to PTSD in a variety of populations including survivors of heart attack (Pedersen et al., 2004), first responders (Ogińska-Bulik and Langer, 2007), and active duty military personnel (Mommersteeg et al., 2011; Rademaker et al., 2011). Recently, Servatius et al. (2017) reported that BI was a significant predictor of PTSD, while Type D was a significant predictor of major depression in active duty Coast Guard personnel. The correlation observed between BI and PTSD in active duty military personnel was consistent with correlations previously observed in Veterans (Myers et al., 2012a).

Taken together, these findings have supported the relationship between PTSD and a variety of constructs describing anxious and inhibited personality temperaments; however, the degree to which trait anxiety, BI, HA, and Type D are distinct constructs has not been adequately addressed. Recent work has revealed that these temperaments have some degree of relationship varying from weak to moderate. Future work needs to examine how much overlap exists between these various measures of inhibited temperament.

In addition to personality temperaments like BI, HA, and Type D, females express greater lifetime prevalence for PTSD than males (Kessler et al., 1995). Partly, this may be due to females having higher likelihood of experiencing certain types of trauma, such as sexual abuse and assault, and/or having higher rates of reporting and seeking treatment (Jacobson et al., 2015). However, at least some studies suggest that the increased risk of PTSD among women is still evident even when exposure to prior traumatic events or history of other pre-existing conditions such as depression or anxiety is controlled for (Breslau et al., 1999). One factor that may help account for the gender differences in PTSD rates may be an interaction with personality temperaments such as BI. Women outscore men on the AMBI in some studies (Gladstone and Parker, 2005; Radell et al., 2016), but not all (Myers et al., 2012a; Allen et al., 2017). The failure to find a clear sex difference could be due to variability in expression of a personality factor (or other vulnerability). For example, Mosing et al. (2012) reported BI temperament to be sex linked in that BI may be more heritable in females than males, which fits with findings of sex-linked genes for HA (Keller et al., 2005). Nonetheless, the additive effect of female gender and BI as well as HA should be recognized to aid in the identification of women who are more likely to develop PTSD.

Overall, several personality temperaments related to avoidance including trait anxiety, behavioral inhibition, harm avoidance, and distressed (Type D) personality have been identified, along with female gender, as vulnerability factors for the development of PTSD. Inhibitory temperaments and a tendency to avoid can possibly identify which trauma-exposed individuals are more at risk to develop PTSD or display resilience, allowing optimal targeting of therapeutic resources.

## An Animal Model of Inhibited Temperament

Besides the strong clinical evidence for a relationship between inhibited temperaments and PTSD, inhibited temperament has been investigated in an animal model, the inbred Wistar Kyoto (WKY) rat strain. WKY rats express many of the behavioral tendencies associated with BI and PTSD in humans. For a detailed review of the WKY rat as an animal model in which pathological avoidance is an endophenotype for PTSD, see Jiao et al. (2011a). Studies have typically compared WKY rats with controls consisting of outbred Sprague Dawley (SD) rats, which do not express inhibited temperaments. WKY rats’ inhibited temperament is evident in several behavioral tendencies including hypervigilance, behavioral withdrawal, avoidance, and hyper-responsivity to stress (Paré, 1989, 1992, 1993; Solberg et al., 2001; Drolet et al., 2002; McAuley et al., 2009; Lemos et al., 2011). WKY rats also express exaggerated hormonal activity (corticosterone and adrenocorticotropic hormone) in the hypothalamic-pituitary axis (HPA) suggesting that WKY rats are inherently hyper-responsive to stressful stimuli, which are similar to findings with behaviorally inhibited children (Smoller et al., 2003, 2005).

WKY rats also exhibit inhibited temperament in a variety of behavioral tasks. One such task is the open field test that measures locomotion and exploration of a novel environment and has been used to test anxiety-like behaviors in rats and mice. Anxiety-related behaviors are thought to come about due to social separation from cage mates and the stress of the novel open field apparatus (File, 1980; Prut and Belzung, 2003). There is evidence that anxiety-like behaviors in the open field test are reduced by GABAergic and serotonergic anxiolytics [see review by Prut and Belzung (2003)]. WKY rats express anxiety-like behaviors in which they are less active and slower to flee the center of the field to the wall at the start of the trial in an open field test (Paré, 1994; Ferguson and Cada, 2004; McAuley et al., 2009) in which a rat is placed in the center of a brightly lit field. The deficit in open field activity has been attributed to anxiety rather than a deficit in locomotion given that WKY rats exhibit normal locomotion in a variety of motor tasks (Paré, 1992; Ferguson and Cada, 2003; Ferguson et al., 2003). The inhibited behavior of WKY rats in the open field test is similar to how behaviorally inhibited individuals express inhibited behaviors in novel social and non-social situations.

Another behavioral tendency of WKY rats is hypervigilance, which fits with the *DSM-5* Criterion E (i.e., alterations in arousal and reactivity) for PTSD. A common measure of hypervigilance is the acoustic startle response (ASR), which tests arousal and vigilance to an unexpected sound. ASR is typically measured by full body movement in rats as compared to an eyeblink response in humans. WKY rats exhibit an enhanced ASR compared to outbred SD rats (Glowa and Hansen, 1994; Servatius et al., 1998; McAuley et al., 2009). There is some evidence of similar enhanced ASR in humans with PTSD (Butler et al., 1990; Morgan et al., 1996). Another test for anxiety is pre-pulse inhibition (PPI) in which a weak stimulus inhibits a subsequent response to a stronger stimulus (usually measured with ASR). Ludewig et al. (2002) reported decreased PPI in humans with panic disorder, while Pineles et al. (2016) found PPI impairments in women with PTSD. However, WKYs exhibited greater pre-pulse inhibition (Conti et al., 2002; McAuley et al., 2009). One possible explanation is that WKY rats may direct attention to the novel, unexpected stimuli by way of hypervigilance, thus resulting in enhanced PPI. In fact, prior studies have shown enhanced PPI when attention is directed to the pre-pulse stimulus (Filion et al., 1993; Ashare et al., 2007). Consistent with this idea, the greater PPI in WKY rats was reduced by the administration of corticotropin releasing factor (CRF), which stimulates the release of adrenocorticotropic hormone (ACTH) as part of the stress response (Conti et al., 2002). Given the relationship between BI and PTSD in humans, the WKY rat may therefore be a model of PTSD vulnerability and anxiety vulnerability.

## Enhanced Avoidance Learning in WKY Rats

Given the behavioral tendency to avoid that is associated with BI in humans, we hypothesized that WKY and SD rats would differ in avoidance learning. In animal models, avoidance takes the form of either passive avoidance (withholding an innate or reflexive response to avoid an aversive stimulus) or active avoidance (performing an arbitrary response to prevent an aversive stimulus). Passive avoidance involves inhibiting innate or species-specific defense responses (SSDRs) such as a preference for a dark area. WKY rats acquire passive avoidance faster than SD rats (Paré, 1993, 1996), which would be expected given that behavioral immobility (i.e., freezing) is a typical coping strategy for the WKY rats. This finding highlights a problem with passive avoidance tasks in which they involve species-specific defense responses or reflexes. Therefore, it is difficult to differentiate passive avoidance learning from modification of naturally occurring fear responses or species-specific defense responses (Bolles, 1970). Based on this issue with passive avoidance, more recent work with avoidance has shifted to active avoidance learning tasks that use an arbitrary response that is learned slowly and incrementally.

Active avoidance in rodents occurs when a voluntary behavior is made in response to a warning signal such as a light or tone in order to avoid an impending aversive event such as an electric foot shock. A variety of behaviors have been tested in recent active avoidance studies in rats including shuttlebox activity (Choi et al., 2010) and stepping onto a platform (Bravo-Rivera et al., 2014), but the behavior that has the longest history of study in avoidance learning is a lever- or bar-press response in which the rat learns to terminate an aversive foot shock by making a lever press, which is termed an escape response. As rats learn the association between the warning signal and the shock, they learn to make a lever press during the warning signal prior to the onset of the shock, which prevents the shock for that trial. This predictive response is termed an avoidance response.

**Figure 1:**
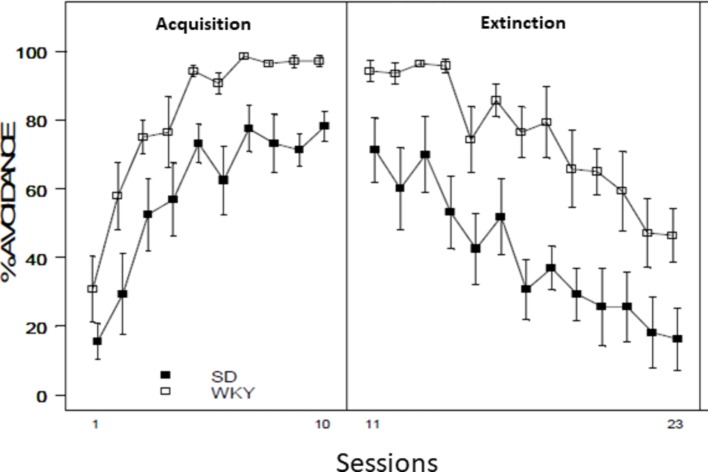
Acquisition and extinction of avoidance responses in response to tone that precedes a foot shock in WKY and SD rats. Behaviorally inhibited WKY rats acquired lever-press avoidance responses faster and to a higher degree than non-inhibited SD controls. When switched to extinction training, WKY rats continue to respond to the tone when the shock is no longer presented early in extinction training, while the SD control rats reduce responding more rapidly. Figure adapted from Jiao et al. (2011a) with permission of the authors.

WKY rats are faster to acquire the lever-press avoidance response and exhibit higher levels of avoidance than SD rats (Servatius et al., 2008; Beck et al., 2010; Jiao et al., 2011b) as shown in Figure 1. In addition, WKY rats exhibit avoidance with non-optimal training situations in which SD rats fail to acquire avoidance (Servatius et al., 2015). For example, although both WKY and SD rats can acquire a lever-press avoidance response when the warning signal is 60 s in duration, WKY rats, but not SD controls, exhibited avoidance with a shorter (10 s) warning signal (Servatius et al., 2015). WKY rats are also more motivated to escape and avoid foot shock than SD rats, even though pain sensitivity to the shock is similar (Fragale et al., 2017; Spiegler et al., 2018). In addition, female WKY rats exhibit enhanced avoidance acquisition and performance as compared to WKY males (Beck et al., 2010, 2011). Therefore, WKY rats exhibit an enhancement of avoidance due to behavioral inhibition, which interacts with female gender and may account for gender differences in expression of PTSD symptoms.

WKY rats exhibit almost perfect avoidance, eventually making an avoidance response on almost every trial. A well-trained WKY rat will then begin each subsequent session with an avoidance response on the first trial and continuing to avoid on all or most trials thereafter. However, SD rats display a different pattern. Even after extensive training, SD rats will typically begin each session with a trial on which they fail to make the avoidance response, experience the shock, and perform an escape response – even though avoidance responses were expressed at high levels at the end of the previous session. This effect is termed warm up (Hineline, 1978a,b) and has been interpreted as SD rats “testing the water” at the start of each session, to see if the old rules still apply (Ricart et al., 2012). Unlike SD rats, WKYs tend to not exhibit warm up. This lack of a warm up effect has been observed in WKY rats, but not SD rats, in eyeblink conditioning (Beck et al., 2011), which suggests that the warm-up effect involves Pavlovian classical conditioning as well as instrumental learning. The tendency of WKY rats to exhibit avoidance responses early in each conditioning session has been linked to worry (Mineka, 2004); the lack of a warm-up effect in WKY rats may indicate that they are performing in the expectation of the impending shock, while the SD rats are performing based on the actual experience of the shock. As a reflection of their hypervigilant temperament, WKY rats predominantly attend to the warning signal compared to the safety signal (Spiegler et al., 2018). By contrast, SD rats focus preferentially on the safety signal rather than the warning signal. Thus, hypervigilance to the warning signal may account for the failure of WKYs to demonstrate the warm-up effect exhibited by SD rats.

The near perfect avoidance behavior of well-trained WKY rats also insulates them from experiencing alterations in shock contingency. For example, when a rat consistently avoids the shock, it cannot become aware of a shift from acquisition trials in which the warning signal is followed by the shock, to extinction training, where warning signal remains but the shock has been removed. Accordingly, WKY rats continue to perform avoidance responses during extinction training, even though the shock is no longer delivered (Servatius et al., 2008; Beck et al., 2011; Jiao et al., 2011b; Perrotti et al., 2013) as shown in Figure 1. However, Beck et al. (2011) reported evidence that WKY rats have a resistance to extinction that goes beyond a lack of experiencing a change in contingency. WKY rats reduced responding by the end of extinction sessions, only to make an avoidance response on the first trial of the next extinction session. Furthermore, Smith et al. (2016) were able to facilitate extinction in WKY rats by housing them in pairs to reduce anxiety. However, while WKYs exhibited facilitated extinction within a session, they continued to make avoidance responses on the first trial of each session. Therefore, WKYs do exhibit a resistance to extinction even after previously reducing avoidance responses to extinction training.

Importantly, these findings of resistance to avoidance extinction fit with the current reports of impaired inhibition of conditioned fear responses in PTSD patients (e.g., Briscione et al., 2014). One of the most successful therapies for PTSD, exposure therapy, is also based on extinction theory. Resistance to extinction has been implicated in the neuropathology of anxiety (Davis and Myers, 2002; Barad, 2005). Overall, WKY rats exhibit several features of avoidance learning, which are consistent with PTSD patients. Specifically, WKY rats exhibit enhanced passive and active avoidance, a worry-like feature, and a resistance to extinction training. These excessive avoidance behaviors may come about due to hypervigilance and over-responding to cues that signal an aversive event which seems similar to the PTSD symptoms of hyperarousal.

## Human Avoidance Learning

Avoidance in humans has not been as widely investigated as in animal models. Some recent human avoidance studies have utilized various aversive events including mild electric shocks (e.g., Lovibond et al., 2008, 2009, 2013; Delgado et al., 2009) and an unpleasant visual or auditory stimulus (e.g., Dymond et al., 2011). For example, Lovibond et al. (2008) used a visual warning signal followed by a delay interval and a period of a possible shock that could be avoided by pressing a specific key. In some cases, these tasks have been designed to include various stages of operant training, Pavlovian classical conditioning, and then a transfer from Pavlovian to instrumental transfer (Lewis et al., 2013). Other studies have included specific instructions to the participants to execute a particular response to terminate or avoid an aversive event (Xia et al., 2017), in which case the task is really assessing the participant’s ability to execute as instructed, rather than assessing behavioral avoidance learning in the manner of the rat lever- press paradigm.

Some studies of human avoidance have employed computer-based avoidance tasks involving a loss of points or loss of monetary reward as the aversive event. For example, Schlund et al. (2011) used a computer-based task in which one stimulus indicated a monetary gain if the correct response is made (i.e., an approach response), while another stimulus indicated a monetary loss that could be avoided if the correct response is made (i.e., an avoidance response). While participants learned to avoid a monetary loss by making the correct response, this avoidance was not the same type as the transition from escape to avoidance responses in the rat lever-press paradigm.

A computer-based avoidance task that may be comparable to escape and avoidance learning in rats was developed by Molet et al. (2006) and adapted by Sheynin et al. (2014a) in which participants controlled a spaceship located at the bottom of the screen. Points were scored by shooting and destroying an enemy spaceship that was moving on the screen. Intermittently, a warning signal occurred that was followed by an aversive event. The aversive event was a bomb that appeared during which the participant’s spaceship exploded and the points were lost. Participants learned to escape or avoid the aversive event by moving their spaceship into a specific “safe area” on the sides of the screen. An escape response was defined as entering the safe area after the bomb period began, which allowed participants to minimize point loss. An avoidance response was defined as entering the safe area during the warning period and remaining there throughout the bomb period, thus avoiding all point loss on that trial. Importantly, subjects received no direct instructions about the function of the safe areas, the appearance of warning signal, or how to escape from or avoid point loss. The vast majority of the participants learned the escape response, while most of them also learned to completely avoid point loss by performing an avoidance response (Sheynin et al., 2014a). This pattern is consistent with what is generally reported in the rodent literature on avoidance learning with the lever- press response as previously discussed (e.g., Servatius et al., 2008). In the Sheynin et al.’s (2014a) study, inhibited individuals (as measured by HA scales) had higher rates of avoidance than uninhibited individuals. In addition, while males and females showed similar rates of avoidance, females avoided for longer periods of time (i.e., on average, females spent more time per trial than males hiding in the safe areas). Sheynin et al. (2014b) extended this task to include an extinction phase following the acquisition phase in which the warning signal was no longer followed by the aversive event (bomb and point loss). As noted above, impaired extinction learning characterizes PTSD and is reflected in patients’ tendency to continue avoiding trauma-associated stimuli, even though the aversive event no longer occurs (Graham and Milad, 2011). Results from the acquisition phase of the spaceship task were similar to those of the prior study (Sheynin et al., 2014a) in which females avoided for longer periods of time than males (Sheynin et al., 2014b). The novel finding from the follow-up study was that females were slower to extinguish the avoidance behavior than males (shown by longer hiding duration during the warning period on extinction trials), an effect that matches the delayed avoidance extinction of WKY rats in the lever-press conditioning paradigm as previously described (Servatius et al., 2008).

**Figure 2:**
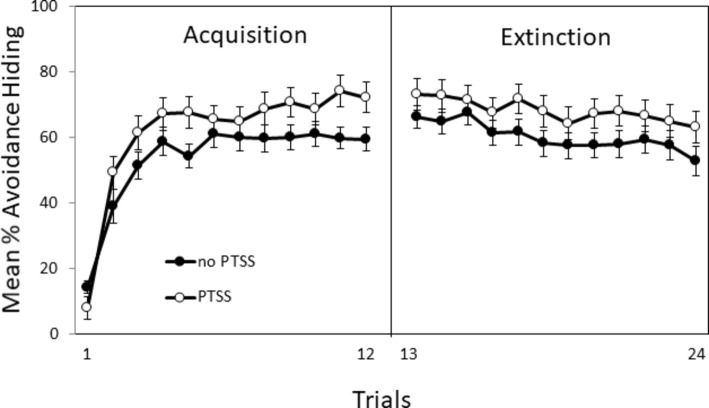
Acquisition and extinction of avoidance responses (hiding during the warning period) in the computer-based task. Individuals with PTSD symptoms (PTSS) exhibited higher levels of avoidance than individuals without PTSD symptoms (noPTSS), but did not differ in avoidance responses during extinction training. Figure adapted from Sheynin et al. (2017) with permission of the authors.

More recently, Sheynin et al. (2017) tested this human avoidance task with Veterans and civilians expressing PTSD symptoms. Individuals with current severe PTSD symptoms (PTSS) exhibited enhanced acquisition of avoidance responses and higher overall levels of avoidance as compared to individuals with few/no PTSD symptoms as shown in Figure 2. Further analysis revealed that the enhanced avoidance was evident mainly in females. Extinction training did not reveal a significant PTSS or gender effect on extinction, which is consistent with the findings of Beck et al. (2011) for the rodent lever-press avoidance task.

Overall, humans expressing inhibited temperament and/or PTSD symptoms exhibit enhanced avoidance learning much like WKY rats. In addition, females exhibited greater avoidance learning than males in both WKY rats and humans. Taken together, the findings from these avoidance studies with WKY rats and humans support the idea that inhibited temperament and female gender are associated with enhanced avoidance and slowed extinction, which may be indicative of PTSD.

## Enhanced Associative Learning and PTSD

In addition to avoidance, several theories of PTSD (Eysenck, 1979; Pitman and Orr, 1986; Pitman, 1988; Rubin et al., 2008; Zuj and Norrholm, 2019) suggest that many PTSD symptoms reflect hyper-conditioning of learned associations in which a cue (a conditioned stimulus or CS) present at the time of the trauma (an unconditioned stimulus or US) comes to evoke a learned emotional response (conditioned response or CR) similar to those provoked by the event itself. According to this model, individual differences in the speed of acquisition of the CR, and in extinction of the CR when the CS is no longer followed by the US, could promote individual differences in the development and maintenance of PTSD symptoms.

Continued over-prediction of aversive stimuli related to a traumatic event can come about through two mechanisms. First, the formation of associations between a cue (the CS) and an aversive event (the US) may be enhanced. For example, a soldier may experience a seemingly neutral event such as a crowd of people (CS) immediately before an aversive event such as an explosion (US). The degree of predictiveness between the neutral and aversive events may be low in which most crowds do not predict upcoming explosions; however, some individuals exposed to one instance of the crowd-explosion association may form stronger than normal associations between the events, such that a future exposure to a crowd may trigger strong memories or feelings associated with the traumatic event (CR). Individuals with PTSD might then avoid similar situations in order to avoid triggering the CR.

Second, the associations formed between a cue and an undesirable stimulus may persist longer than normal. Normally, when cues no longer predict an undesirable event, the association weakens and the behavior is reduced. A real-world example would be a soldier who learned an association between crowds and explosions during periods of combat exposure and then returns home to experience many instances of crowds (shopping mall, movie theater, etc.) with no explosion. The repeated lack of pairing between crowd CS and explosion US would normally lead to extinction of the CS-US association. However, an individual with PTSD might experience crowds many times without the outcome of an explosion and yet continue to experience stress and fear responses related to the traumatic event due to the initial strength of the association between crowds and the traumatic event or an inability to associate crowds with alternative responses, or lack of extinction.

Individual differences in personality temperament (e.g., BI) may produce differences in either the acquisition of conditioned responses through CS-US paired training or the reduction of previously trained responses through CS-alone extinction training, or both. Behaviorally inhibited individuals would form strong associations between traumatic events and trauma-related cues resulting in fear, anxiety, and avoidance in the presence of these cues. These learned responses contribute to dysfunction when expressed pathologically in the absence of danger.

Consistent with this view, some studies utilizing associative learning paradigms with aversive stimuli, such as shock, have reported facilitated acquisition of autonomic (i.e., fear) conditioned responses in humans expressing PTSD symptoms (Grillon et al., 1999; Grillon and Morgan, 1999; Orr et al., 2000; Peri et al., 2000; Blechert et al., 2007; Wessa and Flor, 2007; Jovanovic et al., 2010). However, other studies have found no such effects (Ayers et al., 2003; Orr et al., 2006; Vythilingam et al., 2006; Ginsberg et al., 2008a,b). The variability of results may partially reflect different experimental design, different parameters such as stimulus duration and intensity, and/or populations in which the trauma differs. One area of focus in recent PTSD studies with fear conditioning involves an impairment in the inhibition of previously conditioned fear responses (for reviews, see Briscione et al., 2014; Zuj et al., 2016; Norrholm and Jovanovic, 2018; Zuj and Norrholm, 2019). Specifically, patients with PTSD exhibit a persistence of conditioned fear responding as evidenced by impaired extinction with CS-alone trials, a lack of fear inhibition to safety signals, and over-generalization of conditioned fear to safety signals. Across all of these studies, there is evidence and persistent fear conditioned responding in PTSD. However, if inhibited temperament produces hyper-conditioning, it should be evident even in conditioned responses that do not explicitly invoke fear or trauma-relevant stimuli.

## Classical Eyeblink Conditioning

The most well-studied form of classical conditioning other than fear conditioning involves the conditioning of an eyeblink response. Classical eyeblink conditioning involves the pairing of an initially neutral CS (i.e., a tone or light) with an aversive US (e.g., corneal air puff with humans and rabbits or periorbital shock with rats) that elicits a reflexive eyeblink termed the unconditioned response or UR. Initially, the tone is neutral in which it does not produce an eyeblink response. However, following paired presentations of the tone followed by the air puff, a conditioned eyeblink or CR is exhibited prior to the onset of the air puff.

## Enhanced Classical Eyeblink Conditioning With Anxiety

There is a long history of studying anxiety in classical eyeblink conditioning going back to the 1950s. Janet Taylor Spence (Taylor, 1953) developed the Manifest Anxiety Scale (MAS), a 50-item true/false inventory, which assessed the personality trait of overt or conscious anxiety. Using the MAS, healthy college-aged individuals were classified as high anxiety (upper 75–80% of scores) and low anxiety (lower 20–25% of scores). In a series of studies, high-anxiety individuals were found to exhibit facilitated eyeblink classical conditioning as compared to low-anxiety individuals (Spence and Taylor, 1951, 1953; Taylor, 1951, 1956; Farber and Spence, 1953; Spence and Farber, 1953, 1954; Spence and Beecroft, 1954; Spence and Weyant, 1960; Spence and Spence, 1966). There was also a gender effect with females having higher manifest anxiety scores than males. Spence (1964) hypothesized that individual differences in the degree of stress experienced during the experiment may have contributed to performance differences between high- and low-anxiety individuals. These classic findings of anxiety-facilitated learning are the historical foundation for more recent studies of the effects of anxiety on eyeblink conditioning in rats and humans.

## Enhanced Eyeblink Classical Conditioning With Inhibited Temperament

**Figure 3:**
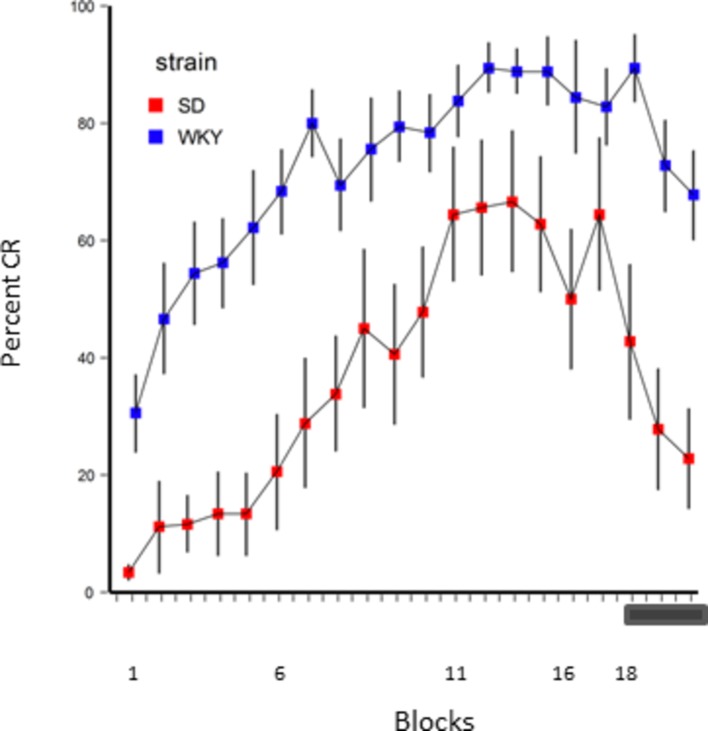
Acquisition of conditioned eyeblinks in WKY and SD rats with a tone CS and eyeshock US. Behaviorally inhibited WKY rats acquired conditioned eyeblinks faster and to a higher degree than non-inhibited SD controls. WKY rats also had slower extinction than SD controls to CS-alone trials (blocks 18–20 as indicated by the gray bar). Figure adapted from data from Beck et al. (2011) with permission of the authors.

Recent work has attempted to determine if the enhanced avoidance in WKY rats and inhibited humans carried over to eyeblink classical conditioning. As expected, WKY rats acquired conditioned eyeblinks faster and to a greater degree than control SD rats (Beck et al., 2011; Ricart et al., 2011b; Janke et al., 2015) as shown in Figure 3. Similar to the early findings from Spence and colleagues, humans expressing trait anxiety (Holloway et al., 2012; Caulfield et al., 2013) and BI (Caulfield et al., 2013) also exhibited faster acquisition of classically conditioned eyeblinks. Caulfield et al. (2013) demonstrated no effect of BI on the reflexive responding to the US, which indicated that increased sensitivity to aversive stimuli could not account for differences in conditioned eyeblink acquisition.

In addition to the enhanced CR acquisition, Beck et al. (2011) reported that WKY rats had slower extinction to CS-alone trials than SD controls as shown in Figure 3. This is similar to the slowed extinction of lever-press avoidance in WKY rats (Servatius et al., 2008; Beck et al., 2011; Jiao et al., 2011b; Perrotti et al., 2013) and the slowed extinction of avoidance responding on the spaceship task in humans (Sheynin et al., 2014b).

More recent work with eyeblink classical conditioning and inhibited personality has extended beyond BI to test the effects of distressed (Type D) personality in non-clinical populations (Allen et al., 2018). As described previously, Type D involves a combination of social inhibition (SI) and negative affectivity (NA). Using the standard cutoff scores for the DS-14 (Denollet, 2005), individuals were categorized as Type D or non-Type D, SI or non-SI, and NA or non-NA. Both Type D and SI individuals expressed enhanced acquisition of conditioned eyeblinks, similar to that previously found for behaviorally inhibited individuals. However, NA individuals did not exhibit any differences in conditioning from non-NA individuals. This finding of spared eyeblink conditioning is in contrast to previous reports of disrupted trace conditioning with depressed individuals (Greer et al., 2005). Further work with Type D and depressed individuals would be of particular interest given the high degree of co-morbidity between PTSD and depression.

## Enhanced Eyeblink Conditioning With Omission

Given the findings of enhanced avoidance and associative learning (i.e., eyeblink classical conditioning) in WKY rats and humans with inhibited temperament, a form of eyeblink conditioning that involves an instrumental avoidance response was also tested. The avoidance version of eyeblink conditioning involves the omission of the US on trials in which a CR is performed to the CS. Early studies of omission training with human eyeblink conditioning revealed that omission training resulted in reduced acquisition compared to 100% CS-US paired trials (Moore and Gormezano, 1961; Gormezano et al., 1962). Furthermore, these studies utilized yoked controls that were presented the same pattern of trials without the US delivery based on the performance of individuals in omission training but without control over US delivery. Yoked controls demonstrated reduced CR performance relative to the omission groups, even though both groups received the same number and pattern of CS trials with the US omitted. Because of the difference between omission and yoked training, it was concluded that participants receiving omission training were acquiring an avoidance response. Therefore, it is possible to test avoidance learning in the context of eyeblink conditioning.

In a rat version of this eyeblink conditioning omission task, the US (eyelid stimulation) was omitted on trials in which the rat performed a CR to the tone (Ricart et al., 2011b). Therefore, if the rat performed a CR, the eye shock was omitted (avoided) for that trial. WKY rats performed better in the US omission tasks than did yoked rats indicating sensitivity of WKY rats to schedules of partial reinforcement. This pattern was not observed in SD controls, which responded similarly to both omission and yoked situations. This pattern that WKY rats, but not SD rats, exhibit eyeblink avoidance fits with the findings of enhanced avoidance for WKY rats in the lever-press avoidance task (Servatius et al., 2008).

The effect of omission training on eyeblink conditioning was also tested with healthy young adults assessed for BI (Holloway et al., 2014). While behaviorally inhibited individuals exhibited enhanced acquisition of eyeblink CRs as compared to non-inhibited individuals, US omission and yoked training did not differ. This finding of no avoidance learning in human eyeblink conditioning differed from that reported for WKY rats by Ricart et al. (2011b). This difference in avoidance learning between omission training in humans and rats may be due in part to the use of a corneal air puff in the human study versus electrical eyelid stimulation in the rat study. The omission of the eyelid stimulation in the rat study may be more salient than the omission of the air puff in the human study in which the aversiveness of the air puff is reduced by the eyelid closure, whereas the aversiveness of the electrical stimulation is presumably unaffected by eyelid closure.

Even though humans did not exhibit avoidance learning, the eyeblink conditioning omission study in humans did lead to a novel finding that the enhanced acquisition of conditioned eyeblinks was more evident in the omission and yoked conditions than in the 100% CS-US paired trials. The finding of enhanced learning in non-optimal learning conditions was theorized to be due to the effects of schedules of partial reinforcement or situations in which some of the trials were tone alone rather than CS-US paired presentations.

## Enhanced Eyeblink Conditioning With Schedules of Partial Reinforcement

Within the context of eyeblink classical conditioning, the US air puff has been interpreted by Leonard and Theios (1967) as a reinforcing event in which presentation of the US reinforces (makes more frequent) the CR. Thus, schedules of partial reinforcement in eyeblink classical conditioning have been defined that involve CS-alone trials that omit the US air puff on some percentage of trials (regardless of whether a CR occurs). In prior work with healthy individuals, schedules of partial reinforcement either reduced CR acquisition as compared to 100% CS-US training (Reynolds, 1958; Ross, 1959; Hartman and Grant, 1960; Ross and Spence, 1960; Runquist, 1963; Perry and Moore, 1965) or did not differ from 100% CS-US training (Humphreys, 1939; Grant et al., 1950; Hake and Grant, 1951; Grant and Schipper, 1952; Moore and Gormezano, 1963; Price et al., 1965; Foth and Runquist, 1970). In addition, schedules of partial reinforcement including CS-alone trials produced a partial reinforcement extinction effect (PREE) in which subsequent extinction was slowed relative to extinction following 100% CS-US paired training (Longenecker et al., 1952; Perry and Moore, 1965; Newman, 1967; Leonard, 1975).

**Figure 4:**
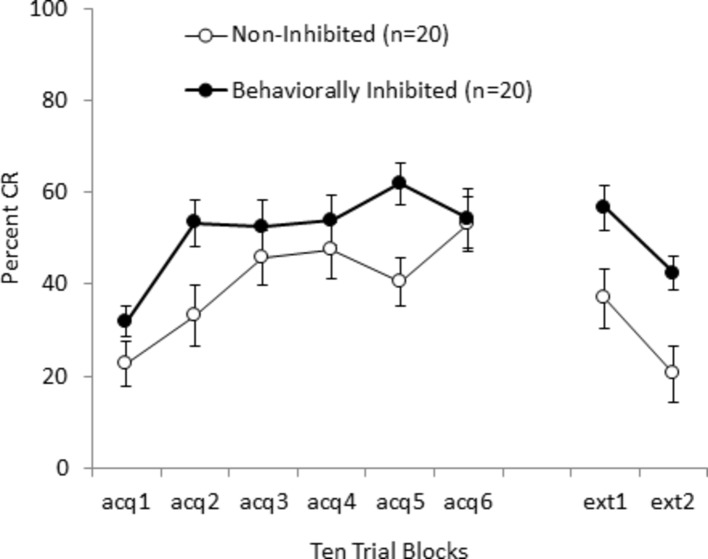
Acquisition and extinction of conditioned eyeblinks in behaviorally inhibited and non-inhibited individuals with a tone CS and air puff US. Behaviorally inhibited individuals acquired conditioned eyeblinks faster and to a greater degree than non-inhibited individuals to a 50% CS-alone schedule of partial reinforcement. When switched to CS-alone extinction training, behaviorally inhibited individuals had slower extinction than non-inhibited individuals. Figure adapted from Allen et al. (2014) with permission of the authors.

The partial reinforcement effect was subsequently tested in humans with BI, using specific schedules of partial reinforcement in which the CS or US were presented alone on 50% of training trials (Allen et al., 2014). Both partial reinforcement schedules enhanced learning of conditioned eyeblinks in behaviorally inhibited individuals as compared to non-inhibited individuals, and the enhancement was even stronger than that apparent with 100% CS-US training which is similar to the findings of Holloway et al. (2014) where omission and yoked training produced greater enhancements than 100% CS-US training. In addition, behaviorally inhibited individuals exhibited slower extinction than non-inhibited individuals when training was switched from 50% CS-alone training to 100% CS-alone extinction training as shown in Figure 4. This slowing of extinction was similar in magnitude to that reported for WKY rats for the avoidance task (Servatius et al., 2008) and in eyeblink classical conditioning (Beck et al., 2011). The finding that extinction was slowed in inhibited animals and humans is important given the use of exposure (i.e., extinction) training for PTSD and anxiety disorders (Joseph and Gray, 2008).

We hypothesized that the enhancement observed in inhibited individuals under partial reinforcement may also have been due in part to the fact that introducing CS- and US-alone trails caused variations in the timing between CS-US paired training trials, compared to the fairly regular presentations of CS-US trails under 100% CS-US training. This hypothesis was tested by extending and varying the inter-trial interval (ITI) between CS-US paired training trials under an 100% CS-US training schedule (Allen et al., 2016). Simply extending the ITI from around 30–60 s did not significantly enhance acquisition; however, extending and varying ITI enhanced CR acquisition – but only in individuals with behavioral inhibition, not their non-inhibited counterparts. This finding may be relevant to PTSD in which uncertainty in when the next trauma-related cue will occur may increase hypervigilance. This finding may also be applicable to determining the most optimal spacing and timing of treatments for PTSD.

## Proactive Interference With Inhibited Temperaments

Another variation of eyeblink conditioning that has been tested with WKY rats and inhibited humans involves the proactive interference effects of stimulus pre-exposure prior to conditioning. Normally, repeated exposures of the CS alone (i.e., latent inhibition or LI), US alone, or uncorrelated presentations of the CS or US (i.e., learned irrelevance) retard subsequent acquisition of conditioned responses. LI is not observed in stressed humans (Braunstein-Bercovitz et al., 2001) or those with trait anxiety (Braunstein-Bercovitz, 2000; Braunstein-Bercovitz et al., 2002). Given these findings and the WKY strain’s enhanced reactivity to stressors, it was hypothesized that LI may be reduced or absent in WKY rats as compared to SD controls. Consistent with this prediction, Ricart et al. (2011a) found that while SD rats exhibited LI following CS alone pre-exposures, WKY rats exhibited normal levels of eyeblink classical conditioning in this task regardless of exposure. In other words, WKY rats’ performance was not disrupted by stimulus pre-exposures that normally would result in a retardation of CR acquisition. This lack of a LI effect in WKY rats may be due to enhanced attention to or vigilance toward aversive stimuli during the conditioning phase. This result is consistent with the finding that WKY rats are hypervigilant and lack the ability to disengage from stimuli as compared to non-inhibited SD rats (McAuley et al., 2009).

However, when Holloway et al. (2012) tested proactive interference in human eyeblink classical conditioning individuals with high-trait anxiety exhibited more proactive interference in response to pre-exposures of the air puff US than individuals with low-trait anxiety. Allen and Miller (2016) also reported that CR acquisition was disrupted in behaviorally inhibited individuals following US alone pre-exposures. These human findings differ from those reported for WKY rats. These inconsistent results may be due to methodological differences between the use of periorbital eye shocks in rats and corneal air puff in humans. Future work should continue to investigate proactive interference effects in rodents and humans, specifically with PTSD symptoms, to determine the effects of stimulus pre-exposure prior to conditioning as a possible pre-emptive treatment to limit PTSD development.

## Enhanced Eyeblink Conditioning in PTSD Populations

In addition to studies of behaviorally inhibited individuals, eyeblink conditioning has also been tested with individuals diagnosed or identified with PTSD symptoms. Myers et al. (2012b) tested the relationship between BI and PTSD symptoms on eyeblink conditioning in a classical conditioning task and an omission training task. Overall, Veterans who self-reported high levels of childhood BI (using the RMBI) had faster acquisition of conditioned eyeblinks than those without childhood BI. As has been found in non-clinical samples, there were no group differences in the amplitude of reflexive eyeblinks, which indicated that the enhanced learning was due to a difference in associative learning rather than differences in reactivity to the stimuli. In addition, Veterans self-reporting current severe PTSD symptoms had slower extinction than those with few/no PTSD symptoms in the omission task; groups did not differ in extinction on the classical conditioning task. This finding is similar to the slowed extinction found in behaviorally inhibited individuals within a schedule of partial reinforcement including CS-alone trials (Allen et al., 2016).

**Figure 5:**
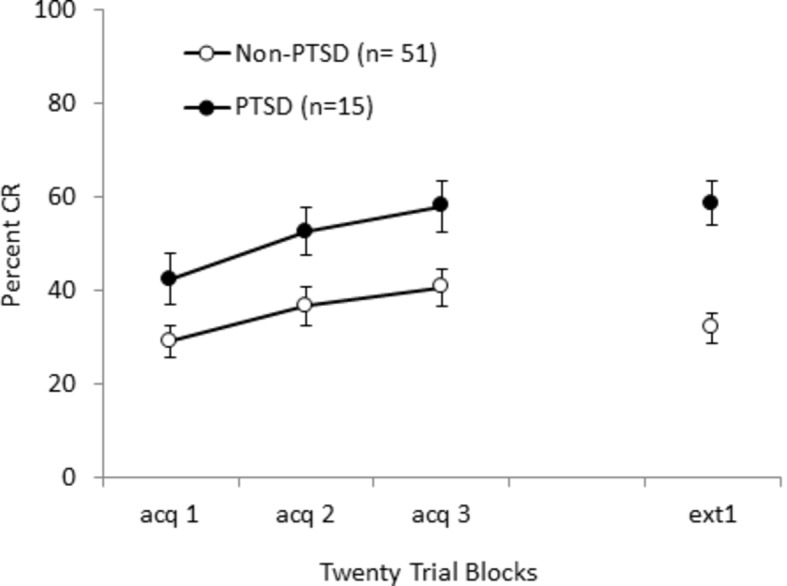
Acquisition and extinction of conditioned eyeblinks with a tone CS and air puff US in military personnel with PTSD symptoms (PTSD+) and without PTSD symptoms (PTSD-). PTSD was associated with faster acquisition to a 50% CS-alone schedule of partial reinforcement and continued responding during extinction (tone alone) training. Figure adapted from Behavioural Brain Research, 339, Handy, J.D., Avcu, P., Ko, N., Ortiz, A., Doria, M.J., and Servatius, R.J. Facilitated acquisition of the classically conditioned eyeblink response in active duty military expressing posttraumatic stress disorder symptoms. 106-113, (2017) with permission of Elsevier.

More recently, Handy et al. (2018) tested BI and PTSD symptoms in active duty Coast Guard personnel, using eyeblink classical conditioning with a 50% CS-alone schedule of partial reinforcement based on the methodology of Allen et al. (2016). Personnel meeting symptom criteria for probable PTSD exhibited more conditioned eyeblinks than non-PTSD personnel as shown in Figure 5. In addition to enhanced acquisition, individuals with probable PTSD symptoms exhibited a pattern of high responsivity to tone-alone extinction trials, which resulted in slower extinction. This finding of slower extinction matches the findings as previously discussed for Veterans with PTSD symptoms in an omission task (Myers et al., 2012a) and in a non-clinical sample trained with 50% CS-alone schedule of partial reinforcement (Allen et al., 2016). Findings of enhanced acquisition and slowed extinction of conditioned eyeblinks with individuals with PTSD symptoms give strong support for the idea that behavioral inhibition, in humans and rodents, is a vulnerability factor that is translated through altered associative learning into PTSD.

**Figure 6:**
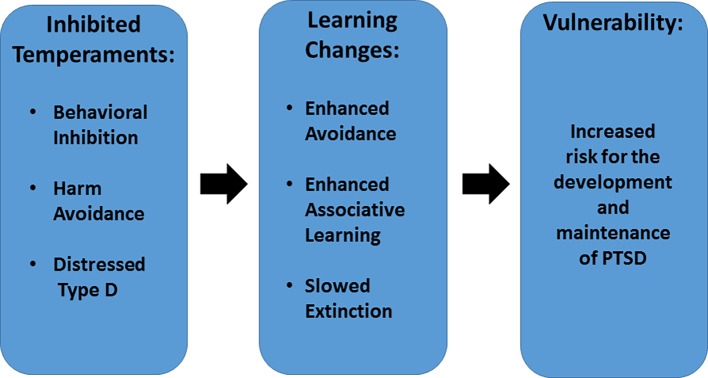
The learning diathesis model of PTSD. Inhibited temperaments interact with changes in avoidance and associative learning leading to increased PTSD vulnerability. This model is supported by the avoidance learning and eyeblink conditioning studies reviewed from the rodent and human literature.

Overall, several measures of anxiety or inhibited temperament including manifest anxiety, trait anxiety, behavioral inhibition, and distressed (Type D) personality have been associated with enhanced associative learning in humans. In addition, individuals with PTSD symptoms also show enhanced associative learning. These findings generally agree with the findings from the behaviorally inhibited WKY rat strain. In addition, these findings support the idea that parallel rodent and human studies provide an opportunity to explore the mechanisms for the development of PTSD through altered associative learning. The work reviewed from WKY rats and inhibited individuals as well as individuals with symptoms of PTSD support for the learning diathesis model of PTSD as represented in Figure 6.

## A Rabbit Eyeblink Model of PTSD

In addition to eyeblink conditioning studies with rats and humans, a separate animal model of PTSD utilizing eyeblink conditioning involves modulation of the eyeblink reflex in rabbits (for review, see Schreurs and Burhans, 2015). In these studies, conditioning with a tone CS and periorbital eye shock US not only produced conditioned eyeblinks (CRs) but also produced a conditioning-specific reflex modulation (CRM) of the UR, specifically an increase in the amplitude and area of the eyeblink response to a periorbital shock; this CRM has been interpreted as reflecting hyperarousal (Burhans et al., 2008). One interesting finding from the CRM rabbit model of PTSD is that only about 25% of rabbits exhibited CRM, even though all rabbits exhibit conditioned eyeblinks (Smith-Bell et al., 2012); this range of outcomes is reminiscent of the fact that only a subset of humans exposed to a trauma develops hypervigilance and other PTSD symptoms. Future work should determine if some aspect of inhibited temperament underlies the individual differences in CRM in rabbits, as well as whether CRM occurs in humans and WKY rats – and if it is associated with inhibited temperament.

## A Role for Uncertainty

One repeated finding from the human eyeblink conditioning studies is that enhanced eyeblink conditioning is most evident in situations that involve some degree of uncertainty. These uncertain conditions – where the CS and US are not always reliably paired – have included the omission and yoked paradigms (Myers et al., 2012a; Holloway et al., 2014), schedules of partial reinforcement (Allen et al., 2014, 2018; Handy et al., 2018), variable trial timing (Allen et al., 2016), and proactive interference tasks where the relationship between the CS and US varies across experimental phases (Holloway et al., 2012). In fact, conditioning protocols that include schedules of partial reinforcement may be more representative of the real world than 100% CS-US paired trials, since real-world events are not as highly controlled as a laboratory conditioning paradigm. The uncertainty inherent in schedules of partial reinforcement may be responsible in part for the enhanced learning in BI and PTSD individuals.

A review of the effects of uncertainty in anxiety identified five mechanisms including inflated estimates of threat, hypervigilance, deficient safety learning, avoidance, and increased reactivity to uncertain threats (Grupe and Nitschke, 2013), which may help explain enhanced avoidance and associative learning in inhibited individuals. Specifically, inflated estimates of threat could underlie the lack of a warm-up effect in which WKY rats respond with an avoidance response from the first trial in a daily session, while non-inhibited SD controls have to receive a few shocks to begin avoiding. An inflated estimate of threat could also account for the enhanced eyeblink classical conditioning observed among WKY rats trained under partial reinforcement. Increased threat attention or hypervigilance could also explain the enhanced avoidance and eyeblink conditioning due to increased activity in the amygdala. Heightened reactivity to uncertainty could play into the enhanced eyeblink conditioning observed in omission and yoked eyeblink conditioning tasks as well as the schedules of partial reinforcement. There is also evidence of deficient safety learning in WKY rats. Beck et al. (2011) tested male and female WKY and SD rats with avoidance learning as previously described with the addition of a safety signal (a flashing light), which was presented during the inter-trial interval (ITI) when no shocks were possible. Male WKY rats were facilitated in avoidance learning by the addition of this safety signal. However, female WKY rats and both male and female SD rats did not exhibit facilitated avoidance with the additional safety signal. However, female WKY rats extinguished faster when the safety signal was removed. Beck et al. (2014) hypothesized that the flashing light during the ITI did not act as a safety signal but rather as an occasion setter by producing a mild increase in arousal or attention to the forthcoming warning signal. In another study suggesting impaired safety signal use in WKY rats, male SD and WKY rats were tested on probe trials after acquisition of the avoidance response (Spiegler et al., 2018). On these probe trials, warning and safety signals were presented together, and SD rats decreased lever presses, as expected if they were attending to the safety signal. By contrast, WKY rats increased lever presses, as predicted if they were responding to the warning signal. The effects of uncertainty could also play into the enhanced behavioral avoidance observed in WKY rats and individuals with BI and/or PTSD. Cognitive avoidance is beyond the scope of the current avoidance and eyeblink conditioning tasks but could be studied in the future. Overall, each of these mechanisms involved with uncertainty might produce enhanced avoidance and/or associative learning in WKY rats and in humans with inhibited temperaments or PTSD.

Uncertainty also plays a role in inhibited temperament and PTSD. Intolerance of uncertainty as measured by the Intolerance of Uncertainty Scale-short version (IUS-12, Carleton et al., 2007) has also been found to be positively correlated to BI and HA (Allen, unpublished data). In addition, Fetzner et al. (2013) found that intolerance of uncertainty was related to all symptoms of PTSD except re-experiencing. Bardeen et al. (2013) reported that intolerance of uncertainty is a moderator between worry and PTSD symptoms. In addition, this relationship was only found for PTSD hyperarousal symptoms. Oglesby et al. (2016) found that pre-trauma intolerance of uncertainty predicted PTSD symptoms following a campus shooting. Uncertainty appears to be a major factor in the learning enhancements observed in the eyeblink conditioning studies, which could shed light on mechanisms for the development of PTSD. Future work in eyeblink conditioning as well as in avoidance learning should include some degree of uncertainty to further explore why individuals with PTSD symptoms are sensitive to uncertainty.

## Overlapping Neural Substrates of Eyeblink Conditioning, Avoidance, and PTSD

The work with avoidance learning and eyeblink classical conditioning in rodents and humans with inhibited temperaments can elucidate the neural substrates through which PTSD develops and is maintained that could be targeted in future therapies. Eyeblink classical conditioning studies involve either delay or trace conditioning. In delay conditioning, the CS precedes and co-terminates with the US, while in trace conditioning, the CS precedes the US but terminates prior to the onset of the US. Thus, there is a trace period between the CS and the US when no stimulus is present. Delay and trace eyeblink conditioning involve different neural systems.

In the case of delay conditioning (which was the task used in the rodent and human classical conditioning studies reviewed in this article), the cerebellum is the substrate of CS-US association (for a detailed review, see Thompson and Steinmetz, 2009). This association can be formed by the cerebellum in the absence of the hippocampus (Schmaltz and Theios, 1972; Gabrieli et al., 1995) and cerebral cortex (Mauk and Thompson, 1987). In trace conditioning, the hippocampus (Moyer et al., 1990; McGlinchey-Berroth et al., 1997; Weiss et al., 1999) and medial prefrontal cortex (Kronforst-Collins and Disterhoft, 1998) are necessary in addition to the cerebellum. However, delay conditioning is modulated by inputs from the septo-hippocampal system (Berry and Thompson, 1979; Allen et al., 2002; Roland et al., 2014) and the amygdala (Blankenship et al., 2005). Additionally, the hippocampus is involved in the enhanced delay conditioning observed in WKY rats (Janke et al., 2015). Enhanced amygdala activity has also been proposed as one mechanism by which BI produces hypervigilance to aversive cues (Allen et al., 2016). The enhanced CR acquisition during delay eyeblink classical conditioning observed in WKY rats, and by humans with BI or PTSD symptoms, might reflect enhanced cerebellar learning and/or modulation of cerebellar learning by hippocampal and amygdala inputs.

There is also some limited evidence that the cerebellum is involved in avoidance learning. Removal of the cerebellum in rats impaired passive avoidance learning in a shuttle box task (Dahhaoui et al., 1990; Guillaumin et al., 1991). More specifically, lesions of the deep cerebellar nuclei prevented learning of active avoidance responses and impaired escape responding in the rat lever-press paradigm (Steinmetz et al., 1993), although the lesions did not disrupt an appetitive version of the task. The lack of an effect on the appetitive version of the task indicates that the lesioned rats were capable of making the motor response (i.e., the lever press) and were able to use the tone as a signal for the availability of a food pellet reward. Therefore, the enhanced avoidance learning observed with WKY rats, and in humans with BI or PTSD symptoms, may also be due to alterations or modulation of learning in the cerebellum.

The possible role of cerebellar learning in PTSD is supported by recent imaging studies that have identified a role for the cerebellum in PTSD (Fernandez et al., 2001; Bonne et al., 2003). Cerebellar hyperactivity has been reported including increased resting state activity (Wang et al., 2016) as well as increased cerebellar blood flow at rest and in response to threat-related stimuli in individuals with PTSD (Bremner et al., 1999, 2003; Osuch et al., 2001; Bonne et al., 2003; Pantazatos et al., 2012). In addition, cerebellar structural abnormalities have been reported in patients with PTSD (Baldaçara et al., 2011; Sussman et al., 2016) including altered connectivity between cerebellum and the cerebral cortex and hippocampus (Rabellino et al., 2018). Overall, there is convergent imaging and clinical evidence that eyeblink classical conditioning, avoidance learning, and PTSD all involve the cerebellum. In recent years, there has been increased attention given to the cerebellum in the context of PTSD and fear- and anxiety-related disorders (for a recent review, see Moreno-Rius, 2018); however, this work has not included behavioral measures, such as eyeblink conditioning or avoidance learning, which are known to involve the cerebellum. The inclusion of these behavioral measures would improve the ongoing work on exploring the role of the cerebellum in the development and maintenance of PTSD.

Beyond the cerebellum, the hippocampus is known to modulate eyeblink conditioning and be involved in avoidance learning as well as PTSD. Although the hippocampus is not required for simple delay eyeblink conditioning, when present it normally contributes to acquisition of the CS-US association (for reviews, see Myers et al., 1996; Moustafa et al., 2013). Disruption of the hippocampus by electrical stimulation or pharmacological manipulation can disrupt acquisition of the eyeblink CR (Berry and Thompson, 1979; Solomon et al., 1983; Allen et al., 2002). In addition, there is some evidence that hippocampal removal or damage accelerates delay classical conditioning (Port et al., 1985) and avoidance learning in shuttle avoidance (Olton, 1973; Black et al., 1977) and lever-press avoidance (Schmaltz and Giulian, 1972; Cominski et al., 2014). There is also evidence for hippocampal abnormalities in WKY rats, which may explain some of aspects their enhanced avoidance learning. WKY rats have reduced hippocampal volume (Lemos et al., 2011; Cominski et al., 2014) as well as impaired hippocampal synaptic plasticity (i.e., long-term potentiation) as compared to SD rats (Cominski et al., 2014). In addition, administration of brain-derived neurotrophic factor (BDNF) into the hippocampus of WKY rats slowed acquisition of eyeblink conditioning to rates similar to SD rats, presumably by enhancing synaptic plasticity (Janke et al., 2015).

These findings of hippocampal abnormalities in WKY rats fit with hippocampal abnormalities observed in humans with PTSD. For example, reduced hippocampal volume is observed in patients with PTSD (Schaefer et al., 2006; Liberzon and Sripada, 2008), along with functional abnormalities observed during fMRI (Werner et al., 2009). In one study, patients with PTSD showed impaired performance on a hippocampal-dependent spatial learning task, and the extent of impairment was correlated with PTSD symptom severity (Astur et al., 2006). Gilbertson et al. (2007) also found that PTSD individuals were impaired at hippocampal-dependent configural learning task. An important question is whether these hippocampal abnormalities precede – and therefore confer vulnerability for – PTSD in individuals subsequently exposed to trauma, or whether these abnormalities emerge as symptoms following trauma exposure and/or development of PTSD. Gilbertson et al. (2002) provided important light on this question in a study documenting reduced hippocampal volume in combat exposed Veterans who developed PTSD as compared to combat-exposed peers who had not developed PTSD; the identical twin brothers of the Veterans with PTSD – who had not been exposed to combat nor developed PTSD – also showed smaller-than-average hippocampi. These data strongly suggest that reduced hippocampal volume may be an individual difference that pre-dates, and confers risk for, PTSD if an individual is subsequently exposed to a traumatic event. Future studies should utilize hippocampal-dependent forms of eyeblink classical conditioning such as trace conditioning to study the hippocampal role in PTSD.

The amygdala also modulates eyeblink conditioning, is involved in avoidance learning, and has been found to be related to PTSD. Specifically, lesions to the amygdala retard acquisition of classically conditioned eyeblinks, disrupt hippocampal activity in rats (Blankenship et al., 2005), and reduce the amplitude of the reflexive eyeblink (UR) in rabbits (Weisz et al., 1992). The amygdala has also been implicated both in the conditioned reflex modification as studied by Schreurs and colleagues in their rabbit model of PTSD as previously discussed (e.g., Schreurs and Burhans, 2015) and in avoidance learning in humans (Simmons et al., 2006; Mobbs et al., 2007; Samanez-Larkin et al., 2008; Straube et al., 2009; Suslow et al., 2009). As previously discussed, the effects of uncertainty in anxiety are thought to come about through enhanced hypervigilance in the amygdala (Grupe and Nitschke, 2013). Furthermore, the medial prefrontal cortex modulates the amygdala and is involved in trace eyeblink conditioning (Kronforst-Collins and Disterhoft, 1998) as well as avoidance extinction (Fragale et al., 2016). In addition, Fragale et al. (2016) reported that the plasticity between the amygdala and the prefrontal cortex is impaired in WKY rats. A lack of inhibition of the amygdala by the medial prefrontal cortex has been theorized to underlie the persistent expression of heightened arousal (e.g., increased acoustic startle) observed in PTSD patients (Nutt and Malizia, 2004).

Overall, there is strong evidence that neural substrates including the cerebellum, hippocampus, amygdala, and medial prefrontal cortex are involved in avoidance learning and eyeblink conditioning as well as PTSD. These brain structures are part of the circuit identified as the cerebellar-limbic-thalamo-cortical network (Schutter, 2013) that serves as an innate alarm circuit and may be altered in cases of PTSD. Behavioral tasks such as avoidance learning and eyeblink conditioning are well suited to further investigate the neural and behavioral mechanisms through which PTSD develops and is expressed.

## Future Work

Future work should focus on further aligning the findings between human studies and animal models of PTSD. Specifically, there is a need for a human avoidance task that more closely matches the trial and error learning aspect as well as the aversive nature of the rat lever-press avoidance paradigm. In addition, studies in WKY rats are needed to replicate recent human findings with schedules of partial reinforcement in both eyeblink conditioning and avoidance learning. To date, eyeblink classical conditioning studies in behaviorally inhibited humans and individuals with PTSD symptoms have been tested exclusively with delay eyeblink conditioning. Future work should also target trace conditioning, which involves the hippocampus and has been found to produce stronger gender differences than delay conditioning (Dalla and Shors, 2009). Future work should also investigate the neural substrates involved in inhibited temperament, as it relates to avoidance and eyeblink conditioning. Additional studies of the neural substrates of WKY rats can identify the mechanisms of inhibited temperament associated with enhanced learning. Functional imaging of behaviorally inhibited individuals and PTSD patients with avoidance learning and eyeblink conditioning should target the cerebellum, hippocampus, amygdala, and medial prefrontal cortex.

## Clinical Applications

Future work can also have potential clinical applications. Avoidance and classical conditioning tasks could be used as pre-tests for PTSD vulnerability in trauma-exposed individuals. These objective measures may be better than standard paper and pencil self-report inventories, which are susceptible to biased responding as well as to individual variations in the ability to introspect and describe one’s own feelings and behaviors in comparison to accepted social norms. If successful, such testing would offer an opportunity for identifying high-risk individuals for a pre-emptive intervention to possibly reduce or avert PTSD before it develops. If the mechanisms through which BI interacts with stimulus pre-exposure could be fully delineated, possible therapies could be developed in which individuals are “inoculated” for PTSD prior to traumatic experiences – similar to the concept of “inoculating” an individual against learned helplessness by training *via* a series of small challenges that can be overcome (e.g., Prindaville and Stein, 1978).

The work with extinction in avoidance learning and eyeblink conditioning is applicable to both identifying individuals at risk for PTSD as well as therapies to reduce PTSD symptoms. Prior work with fear conditioning has indicated that pre-trauma differences in classical conditioning (Orr et al., 2012) and extinction (Guthrie and Bryant, 2006; Lommen et al., 2013) are potential risk factors for PTSD (for a review, see Zuj et al., 2016). These findings fit with our findings of enhanced eyeblink conditioning and slowed extinction in non-clinical samples of behaviorally inhibited individuals. Already, extinction therapy is one of the two major approved therapies approved for PTSD by the Veterans Administration. A better understanding of why, exactly, WKY rats and behaviorally inhibited humans as well as individuals with PTSD have trouble extinguishing might allow us to improve the training schedule in a course of prolonged exposure therapy, thus improving outcomes. Equally important would be the study of why some individuals are resilient following a trauma and do not develop PTSD and are able to extinguish more easily than those individuals who do develop PTSD. These insights into resilience could be applied to help patients with PTSD extinguish their own trauma-related associations. The WKY rat model of PTSD is also well suited for testing pharmacological treatments for PTSD. For example, Jiao et al. (2014) tested the effectiveness of dopaminergic and noradrenergic manipulations to alter acquisition and extinction of avoidance responses. Overall, avoidance and associative learning can be valuable tools in animal and human models of PTSD for the identification of individuals at risk for development of PTSD as well as treatment of PTSD.

## Summary

The exact mechanisms through which a traumatic event leads a limited number of individuals to develop of PTSD, while others develop resilience, remain elusive. What is abundantly clear is that PTSD is best explained with diathesis models (Zinbarg and Barlow, 1996; Kendler et al., 2002; Mineka and Zinbarg, 2006) that address pre-existing vulnerabilities. In the work reviewed here, we have presented support for a learning diathesis model in which inhibited personality temperaments, which are risk factors for PTSD, are associated with enhanced avoidance learning and classical eyeblink conditioning. This evidence has come from parallel studies in a WKY rat model of inhibited temperament as well as from human clinical and non-clinical populations. Both of these learning tasks are of interest given a renewed interest in avoidance learning (e.g., LeDoux et al., 2017; Pittig et al., 2018) and recent imaging studies of cerebellar substrates of PTSD (e.g., Moreno-Rius, 2018). The combination of continued parallel human and non-human studies with these learning tasks holds the potential for increasing our understanding of how inhibited personality temperaments can be translated through enhanced learning into increasing the risk for the development and maintenance of PTSD.

## Author Contributions

MA wrote the original manuscript based on the prior publications of MA, CM, KB, KP, and RS. CM, KB, KP, and RS revised the manuscript.

### Conflict of Interest Statement

The authors declare that the research was conducted in the absence of any commercial or financial relationships that could be construed as a potential conflict of interest.

The contents do not necessarily reflect the views of the U.S. Department of Veterans Affairs or the United States Government.

## References

[ref1] Allen M. T. Miller D. P. (2016). Enhanced eyeblink conditioning in behaviorally inhibited individuals is disrupted by proactive interference following US alone pre-exposures. Front. Behav. Neurosci. 10:39. 10.3389/fnbeh.2016.00039 27014001 PMC4785178

[ref2] Allen M. T. Padilla Y. Gluck M. A. (2002). Ibotenic acid lesions of the medial septum retard delay eyeblink conditioning in rabbits. Behav. Neurosci. 116, 733–738. 10.1037/0735-7044.116.4.733, PMID: 12148941

[ref3] Allen M. T. Myers C. E. Servatius R. J. (2014) Avoidance prone individuals self-reporting behavioral inhibition exhibit facilitated acquisition and altered extinction of conditioned eyeblinks with partial reinforcement schedules. Front. Behav. Neurosci. 8:347. 10.1016/S2215-0366(14)70324-4, PMID: 25339877 PMC4186341

[ref4] Allen M. T. Myers C. E. Servatius R. J. (2016). Uncertainty of trial timing enhances acquisition of conditioned eyeblinks in anxiety vulnerable individuals. Behav. Brain Res. 304, 86–91. 10.1016/j.bbr.2016.02.007, PMID: 26873040

[ref5] Allen M. T. Jameson M. M. Myers C. E. (2017). Beyond behavioral inhibition, a computer avatar task designed to assess behavioral inhibition extends to harm avoidance. Front. Psychol. 8:1560. 10.3389/fpsyg.2017.01560 28966600 PMC5605618

[ref6] Allen M. T. Handy J. D. Blankenship M. R. Servatius R. J. (2018). The distressed (Type D) personality factor of social inhibition, but not negative affectivity, enhances eyeblink conditioning. Behav. Brain Res. 345, 93–103. 10.1016/j.bbr.2018.02.035, PMID: 29486267

[ref7] American Psychiatric Association (2013). Diagnostic and statistical manual of mental disorders. 5th edn. Washington, D.C.: American Psychiatric Publication.

[ref8] Amstadter A. B. Nugent N. R. Koenen K. C. (2009). Genetics of PTSD: fear conditioning as a model for future research. Psychiatr. Ann. 39:358. 10.3928/00485713-20090526-01, PMID: 19779593 PMC2749314

[ref9] Ashare R. L. Hawk L. W. Mazzullo R. J. (2007). Motivated attention: incentive effects on attentional modification of prepulse inhibition. Psychophysiology 44, 839–845. 10.1111/j.1469-8986.2007.00563.x, PMID: 17640265 PMC2650018

[ref10] Astur R. S. St Germain S. A. Tolin D. Ford J. Russell D. Stevens M. (2006). Hippocampus function predicts severity of post-traumatic stress disorder. Cyberpsychol. Behav. 9, 234–240. 10.1089/cpb.2006.9.234, PMID: 16640486

[ref11] Ayers E. D. White J. Powell D. A. (2003). Pavlovian eyeblink conditioning in combat veterans with and without post-traumatic stress disorder. Integr. Physiol. Behav. Sci. 38, 230–247. 10.1007/BF02688856 15070085

[ref12] Baldaçara L. Jackowski A. P. Schoedl A. Pupo M. Andreoli S. B. Mello M. F.. (2011). Reduced cerebellar left hemisphere and vermal volume in adults with PTSD from a community sample. J. Psychiatr. Res. 45, 1627–1633. 10.1016/j.jpsychires.2011.07.013, PMID: 21824628

[ref13] Barad M. (2005). Fear extinction in rodents: basic insight to clinical promise. Curr. Opin. Neurobiol. 15, 710–715. 10.1016/j.conb.2005.10.005, PMID: 16260129

[ref14] Bardeen J. R. Fergus T. A. Wu K. D. (2013). The interactive effect of worry and intolerance of uncertainty on posttraumatic stress symptoms. Cogn. Ther. Res. 37, 742–751. 10.1007/s10608-012-9512-1

[ref15] Barlow D. H. (2002). Anxiety and its disorders. 2nd edn. New York: Guilford Press.

[ref16] Beck K. D. Jiao X. Pang K. C. Servatius R. J. (2010). Vulnerability factors in anxiety determined through differences in active-avoidance behavior. Prog. Neuropsychopharmacol. Biol. Psychiatry 34, 852–860. 10.1016/j.pnpbp.2010.03.036, PMID: 20382195

[ref17] Beck K. D. Jiao X. Ricart T. M. Myers C. E. Minor T. R. Pang K. C.. (2011). Vulnerability factors in anxiety: strain and sex differences in the use of signals associated with non-threat during the acquisition and extinction of active-avoidance behavior. Prog. Neuropsychopharmacol. Biol. Psychiatry 35, 1659–1670. 10.1016/j.pnpbp.2011.05.002, PMID: 21601608

[ref18] Beck K. D. Jiao X. Smith I. M. Myers C. E. Pang K. C. Servatius R. J. (2014). ITI-Signals and prelimbic cortex facilitate avoidance acquisition and reduce avoidance latencies, respectively, in male WKY rats. Front. Behav. Neurosci. 8:403. 10.3389/fnbeh.2014.00403, PMID: 25484860 PMC4240176

[ref19] Berry S. Thompson R. (1979). Medial septal lesions retard classical conditioning of the nictitating membrane response in rabbits. Science 205, 209–211. 10.1126/science.451592, PMID: 451592

[ref20] Biederman J. Rosenbaum J. F. Bolduc-Murphy E. A. Faraone S. V. Chaloff J. Hirshfeld D. R. (1993). A 3-year follow-up of children with and without behavioral inhibition. J. Am. Acad. Child Adolesc. Psychiatry 32, 814–821. 8340303 10.1097/00004583-199307000-00016

[ref21] Binder E. B. Bradley R. G. Liu W. Epstein M. P. Deveau T. C. Mercer K. B.. (2008). Association of FKBP5 polymorphisms and childhood abuse with risk of posttraumatic stress disorder symptoms in adults. JAMA 299, 1291–1305. 10.1001/jama.299.11.1291, PMID: 18349090 PMC2441757

[ref22] Black A. H. Nadel L. O’Keefe J. (1977). Hippocampal function in avoidance learning and punishment. Psychopharmacol. Bull. 84, 1107–1129. 10.1037/0033-2909.84.6.1107, PMID: 928572

[ref23] Blankenship M. R. Huckfeldt R. Steinmetz J. J. Steinmetz J. E. (2005). The effects of amygdala lesions on hippocampal activity and classical eyeblink conditioning in rats. Brain Res. 1035, 120–130. 10.1016/j.brainres.2004.11.061, PMID: 15722052

[ref24] Blechert J. Michael T. Vriends N. Margraf J. Wilhelm F. H. (2007). Fear conditioning in posttraumatic stress disorder: evidence for delayed extinction of autonomic, experiential, and behavioural responses. Behav. Res. Ther. 45, 2019–2033. 10.1016/j.brat.2007.02.012, PMID: 17442266

[ref25] Bögels S. M. Mansell W. (2004). Attention processes in the maintenance and treatment of social phobia: hypervigilance, avoidance and self-focused attention. Clin. Psychol. Rev. 24, 827–856. 10.1016/j.cpr.2004.06.005 15501558

[ref26] Bolles R. C. (1970). Species-specific defense reactions and avoidance learning. Psychol. Rev. 77, 32–48. 10.1037/h0028589

[ref27] Bonne O. Gilboa A. Louzoun Y. Brandes D. Yona I. Lester H.. (2003). Resting regional cerebral perfusion in recent posttraumatic stress disorder. Biol. Psychiatry 54, 1077–1086. 10.1016/S0006-3223(03)00525-0, PMID: 14625150

[ref28] Braunstein-Bercovitz H. (2000). Is the attentional dysfunction in schizotypy related to anxiety? Schizophr. Res. 46, 255–267. 10.1016/S0920-9964(00)00021-9, PMID: 11120437

[ref29] Braunstein-Bercovitz H. Dimentman-Ashkenazi I. Lubow R. E. (2001). Stress affects the selection of relevant from irrelevant stimuli. Emotion 1, 182–192. 10.1037/1528-3542.1.2.182, PMID: 12899196

[ref30] Braunstein-Bercovitz H. Rammsayer T. Gibbons H. Lubow R. E. (2002). Latent inhibition deficits in high-schizotypal normals: symptom-specific or anxiety-related? Schizophr. Res. 53, 109–121. 10.1016/S0920-9964(01)00166-9, PMID: 11728844

[ref31] Bravo-Rivera C. Roman-Ortiz C. Brignoni-Perez E. Sotres-Bayon F. Quirk G. J. (2014). Neural structures mediating expression and extinction of platform-mediated avoidance. J. Neurosci. 34, 9736–9742. 10.1523/JNEUROSCI.0191-14.2014, PMID: 25031411 PMC4099548

[ref32] Bremner J. D. Narayan M. Staib L. H. Southwick S. M. McGlashan T. Charney D. S. (1999). Neural correlates of memories of childhood sexual abuse in women with and without posttraumatic stress disorder. Am. J. Psychiatry 156, 1787–1795. 10.1176/ajp.156.11.1787, PMID: 10553744 PMC3233772

[ref33] Bremner J. D. Vythilingam M. Vermetten E. Southwick S. M. McGlashan T. Staib L. H.. (2003). Neural correlates of declarative memory for emotionally valenced words in women with posttraumatic stress disorder related to early childhood sexual abuse. Biol. Psychiatry 53, 879–889. 10.1016/S0006-3223(02)01891-7, PMID: 12742675

[ref34] Breslau N. Kessler R. C. Chilcoat H. D. Schultz L. R. Davis G. C. Andreski P. (1998). Trauma and posttraumatic stress disorder in the community: the 1996 Detroit Area Survey of Trauma. Arch. Gen. Psychiatry 55, 626–632. 10.1001/archpsyc.55.7.626, PMID: 9672053

[ref35] Breslau N. Chilcoat H. D. Kessler R. C. Peterson E. L. Lucia V. C. (1999). Vulnerability to assaultive violence: further specification of the sex difference in post-traumatic stress disorder. Psychol. Med. 29, 813–821. 10.1017/S0033291799008612, PMID: 10473308

[ref36] Briscione M. A. Jovanovic T. Norrholm S. D. (2014). Conditioned fear associated phenotypes as robust, translational indices of trauma-, stressor-, and anxiety-related behaviors. Front. Psychiatry 5:88. 10.3389/fpsyt.2014.00088, PMID: 25101010 PMC4104832

[ref37] Burhans L. B. Smith-Bell C. Schreurs B. G. (2008). Conditioning-specific reflex modification of the rabbit’s nictitating membrane response and heart rate: behavioral rules, neural substrates, and potential applications to posttraumatic stress disorder. Behav. Neurosci. 122, 1191–1206. 10.1037/a0013599, PMID: 19045939

[ref38] Butler R. W. Braff D. L. Rausch J. L. Jenkins M. A. Sprock J. Geyer M. A. (1990). Physiological evidence of exaggerated startle response in a subgroup of Vietnam veterans with combat-related PTSD. Am. J. Psychiatry 147, 1308–1312. 10.1176/ajp.147.10.1308, PMID: 2399998

[ref39] Carleton R. N. Norton M. P. J. Asmundson G. J. (2007). Fearing the unknown: a short version of the intolerance of uncertainty scale. J. Anxiety Disord. 21, 105–117. 10.1016/j.janxdis.2006.03.014, PMID: 16647833

[ref40] Casada J. H. Roache J. D. (2005). Behavioral inhibition and activation in posttraumatic stress disorder. J. Nerv. Ment. Dis. 193, 102–109. 10.1097/01.nmd.0000152809.20938.37, PMID: 15684912

[ref41] Casada J. H. Roache J. D. (2006). Dissociation of physiology and behavior in PTSD. Int. J. Psychophysiol. 62, 243–248. 10.1016/j.ijpsycho.2006.04.005, PMID: 16814888

[ref42] Caulfield M. D. McAuley J. D. Servatius R. J. (2013). Facilitated acquisition of eyeblink conditioning in those vulnerable to anxiety disorders. Front. Hum. Neurosci. 7:348. 10.3389/fnhum.2013.00348, PMID: 23847516 PMC3701872

[ref43] Chang C. M. Lee L. C. Connor K. M. Davidson J. R. Jeffries K. Lai T. J. (2003). Posttraumatic distress and coping strategies among rescue workers after an earthquake. J. Nerv. Ment. Dis. 191, 391–398. 10.1097/01.NMD.0000071588.73571.3D 12826921

[ref44] Charlton P. F. C. Thompson J. A. (1996). Ways of coping with psychological distress after trauma. Br. J. Clin. Psychol. 35, 517–530. 10.1111/j.2044-8260.1996.tb01208.x, PMID: 8955538

[ref45] Choi J. S. Cain C. K. LeDoux J. E. (2010). The role of amygdala nuclei in the expression of auditory signaled two-way active avoidance in rats. Learn. Mem. 17, 139–147. 10.1101/lm.1676610, PMID: 20189958 PMC2832923

[ref46] Cloninger C. R. Przybeck T. R. Svrakic D. M. (1991). The tridimensional personality questionnaire: U.S. normative data. Psychiatr. Rep. 69, 1047–1057. 10.2466/pr0.1991.69.3.1047 1784653

[ref47] Cloninger C. R. Svrakic D. M. Przybeck T. R. (1993). A psychobiological model of temperament and character. Arch. Gen. Psychiatry 50, 975–990. 10.1001/archpsyc.1993.01820240059008, PMID: 8250684

[ref48] Cominski T. P. Jiao X. Catuzzi J. E. Stewart A. L. Pang K. C. (2014). The role of the hippocampus in avoidance learning and anxiety vulnerability. Front. Behav. Neurosci. 8:273. 10.3389/fnbeh.2014.00273, PMID: 25152721 PMC4125878

[ref49] Conti L. H. Murry J. D. Ruiz M. A. Printz M. P. (2002). Effects of corticotropin-releasing factor on prepulse inhibition of the acoustic startle response in two rat strains. Psychopharmacology 161, 296–303. 10.1007/s00213-002-1025-2, PMID: 12021833

[ref50] Dahhaoui M. Caston J. Auvray N. Reber A. (1990). Role of the cerebellum in an avoidance conditioning task in the rat. Physiol. Behav. 47, 1175–1180. 10.1016/0031-9384(90)90369-F, PMID: 2395922

[ref51] Dalla C. Shors T. J. (2009). Sex differences in learning processes of classical and operant conditioning. Physiol. Behav. 97, 229–238. 10.1016/j.physbeh.2009.02.035, PMID: 19272397 PMC2699937

[ref52] Davidson J. R. T. (2000). Trauma: the impact of post-traumatic stress disorder. J. Psychopharmacol. 14(2_suppl. 1), S5–S12. 10.1177/02698811000142S102, PMID: 10888026

[ref53] Davis M. Myers K. M. (2002). The role of glutamate and gamma-aminobutyric acid in fear extinction: clinical implications for exposure therapy. Biol. Psychiatry 52, 998–1007. 10.1016/S0006-3223(02)01507-X, PMID: 12437940

[ref54] Delgado M. R. Jou R. L. Ledoux J. E. Phelps E. A. (2009). Avoiding negative outcomes: tracking the mechanisms of avoidance learning in humans during fear conditioning. Front. Behav. Neurosci. 3:33. 10.3389/neuro.08.033.2009, PMID: 19847311 PMC2762377

[ref55] Denollet J. (2005). DS-14: standard assessment of negative affectivity, social inhibition, and Type D personality. Psychosom. Med. 67, 89–97. 10.1097/01.psy.0000149256.81953.49, PMID: 15673629

[ref56] DiGrande L. Perrin M. A. Thorpe L. E. Thalji L. Murphy J. Wu D.. (2008). Posttraumatic stress symptoms, PTSD, and risk factors among lower Manhattan residents 2-3 years after the September 11, 2001 terrorist attacks. J. Trauma. Stress 21, 264–273. 10.1002/jts.20345, PMID: 18553414

[ref57] Drolet G. Proulx K. Pearson D. Rochford J. Deschepper C. F. (2002). Comparisons of behavioral and neurochemical characteristics between WKY, WKHA, and Wistar rat strains. Neuropsychopharmacology 27, 400–409. 10.1016/S0893-133X(02)00303-2, PMID: 12225697

[ref58] Dymond S. Schlund M. W. Roche B. Whelan R. Richards J. Davies C. (2011). Inferred threat and safety: symbolic generalization of human avoidance learning. Behav. Res. Ther. 49, 614–621. 10.1016/j.brat.2011.06.007, PMID: 21767825

[ref59] Engelhard I. M. Van Den Hout M. A. Schouten E. G. (2006). Neuroticism and low educational level predict the risk of posttraumatic stress disorder in women after miscarriage or stillbirth. Gen. Hosp. Psychiatry 28, 414–417. 10.1016/j.genhosppsych.2006.07.001, PMID: 16950377

[ref60] Eysenck H. (1979). The conditioning model of neurosis. Behav. Brain Sci. 2, 155–199.

[ref61] Eysenck M. W. Calvo M. G. (1992). Anxiety and performance: the processing efficiency theory. Cogn. Emot. 6, 409–434. 10.1080/02699939208409696

[ref62] Farber I. E. Spence K. W. (1953). Complex learning and conditioning as a function of anxiety. J. Exp. Psychol. 45, 120–125. 10.1037/h0063618, PMID: 13052840

[ref500] Ferguson S. A. Cada A. M. (2003). A longitudinal study of short- and long-term activity levels in male and female spontaneously hypertensive, Wistar-Kyoto, and Sprague-Dawley rats. Behav. Neurosci. 117, 271–282. 10.1037/0735-7044.117.2.271, PMID: 12708524

[ref63] Ferguson S. A. Cada A. M. (2004). Spatial learning/memory and social and nonsocial behaviors in the spontaneously hypertensive, Wistar–Kyoto and Sprague–Dawley rat strains. Pharmacol. Biochem. Behav. 77, 583–594. 10.1016/j.pbb.2003.12.014, PMID: 15006470

[ref64] Ferguson S. A. Gray E. P. Cada A. M. (2003). Early behavioral development in the spontaneously hypertensive rat: a comparison with the Wistar-Kyoto and Sprague-Dawley strains. Behav. Neurosci. 117, 263–270. 10.1037/0735-7044.117.2.263, PMID: 12708523

[ref65] Fernandez M. Pissiota A. Frans Ö. von Knorring L. Fischer H. Fredrikson M. (2001). Brain function in a patient with torture related post-traumatic stress disorder before and after fluoxetine treatment: a positron emission tomography provocation study. Neurosci. Lett. 297, 101–104. 10.1016/S0304-3940(00)01674-8, PMID: 11121880

[ref66] Fetzner M. G. Horswill S. C. Boelen P. A. Carleton R. N. (2013). Intolerance of uncertainty and PTSD symptoms: exploring the construct relationship in a community sample with a heterogeneous trauma history. Cogn. Ther. Res. 37, 725–734. 10.1007/s10608-013-9531-6

[ref67] File S. E. (1980). The use of social interaction as a method for detecting anxiolytic activity of chlordiazepoxide-like drugs. J. Neurosci. Methods 2, 219–238. 10.1016/0165-0270(80)90012-6, PMID: 6120260

[ref68] Filion D. L. Dawson M. E. Schell A. M. (1993). Modification of the acoustic startle-reflex eyeblink: a tool for investigating early and late attentional processes. Biol.Psychol. 35, 185–200. 10.1016/0301-0511(93)90001-O, PMID: 8218613

[ref69] Fincham D. Smit J. Carey P. Stein D. J. Seedat S. (2008). The relationship between behavioural inhibition, anxiety disorders, depression and CD4 counts in HIV-positive adults: a cross-sectional controlled study. AIDS Care 20, 1279–1283. 10.1080/09540120801927025, PMID: 19012085

[ref71] Foth D. L. Runquist W. N. (1970). Effects of unconditioned stimulus intensity and schedules of 50% partial reinforcement in human classical eyelid conditioning. J. Exp. Psychol. 84, 244–247. 10.1037/h0029087, PMID: 5480924

[ref72] Fragale J. E. Khariv V. Gregor D. M. Smith I. M. Jiao X. Elkabes S. (2016). Dysfunction in amygdala-prefrontal plasticity and extinction-resistant avoidance: a model for anxiety disorder vulnerability. Exp. Neurol. 275, 59–68. 10.1016/j.expneurol.2015.11.002 26546833 PMC4791945

[ref73] Fragale J. E. Beck K. D. Pang K. C. (2017). Use of the exponential and exponentiated demand equations to assess the behavioral economics of negative reinforcement. Front. Neurosci. 11:77. 10.3389/fnins.2017.00077, PMID: 28270744 PMC5318419

[ref74] Gabrieli J. D. McGlinchey-Berroth R. Carrillo M. C. Gluck M. A. Cermak L. S. Disterhoft J. F. (1995). Intact delay-eyeblink classical conditioning in amnesia. Behav. Neurosci. 109, 819–827. 10.1037/0735-7044.109.5.819, PMID: 8554707

[ref75] Galatzer-Levy I. R. Bryant R. A. (2013). 636,120 ways to have posttraumatic stress disorder. Perspect. Psychol. Sci. 8, 651–662. 10.1177/1745691613504115 26173229

[ref76] Gil S. (2005). Pre-traumatic personality as a predictor of post-traumatic stress disorder among undergraduate students exposed to a terrorist attack: a prospective study in Israel. Pers. Individ. Differ. 39, 819–827. 10.1016/j.paid.2005.03.007

[ref77] Gil S. Caspi Y. (2006). Personality traits, coping style, and perceived threat as predictors of posttraumatic stress disorder after exposure to a terrorist attack: a prospective study. Psychosom. Med. 68, 904–909. 10.1097/01.psy.0000242124.21796.f8, PMID: 17079704

[ref78] Gilbertson M. Shenton M. Ciszewski A. Kasai K. Lasko N. Orr S. (2002). Smaller hippocampal volume predicts pathologic vulnerability to psychological trauma. Nat. Neurosci. 5, 1111–1113. 10.1038/nn958 12379862 PMC2819093

[ref79] Gilbertson M. W. Williston S. K. Paulus L. A. Lasko N. B. Gurvits T. V. Shenton M. E.. (2007). Configural cue performance in identical twins discordant for posttraumatic stress disorder: theoretical implications for the role of hippocampal function. Biol. Psychiatry 62, 513–520. 10.1016/j.biopsych.2006.12.023, PMID: 17509537 PMC2768050

[ref80] Ginsberg J. P. Ayers E. Burriss L. Powell D. A. (2008a). Disruption of bradycardia associated with discriminative conditioning in combat veterans with PTSD. Neuropsychiatr. Dis. Treat 4, 635–646. 10.2147/NDT.S2808 18830395 PMC2526370

[ref81] Ginsberg J. P. Ayers E. Burriss L. Powell D. A. (2008b). Discriminative delay Pavlovian eyeblink conditioning in veterans with and without posttraumatic stress disorder. J. Anxiety Disord. 22, 809–823. 10.1016/j.janxdis.2007.08.009 17913453

[ref82] Gladstone G. L. Parker G. (2005). Measuring a behaviorally inhibited temperament style: development and initial validation of new self-report measure. Psychiatry Res. 135, 133–143. 10.1016/j.psychres.2005.03.005, PMID: 15922458

[ref83] Gladstone G. L. Parker G. B. Mitchell P. B. Wilhelm K. A. Malhi G. S. (2005). Relationship between self-reported childhood behavioral inhibition and lifetime anxiety disorders in a clinical sample. Depress. Anxiety 22, 103–113. 10.1002/da.20082, PMID: 16149043

[ref84] Glowa J. R. Hansen C. T. (1994). Differences in response to an acoustic startle stimulus among forty-six rat strains. Behav. Genet. 24, 79–84. 10.1007/BF01067931, PMID: 8192623

[ref85] Gormezano I. Moore J. W. Deaux E. (1962). Supplementary report: yoked comparisons of classical and avoidance eyelid conditioning under 3 UCS intensities. J. Exp. Psychol. 64, 551–552. 10.1037/h0047193, PMID: 13949313

[ref86] Gould T. D. Gottesman I. I. (2006). Psychiatric endophenotypes and the development of valid animal models. Genes Brain Behav. 5, 113–119. 10.1111/j.1601-183X.2005.00186.x, PMID: 16507002

[ref87] Graham B. M. Milad M. R. (2011). The study of fear extinction: implications for anxiety disorders. Am. J. Psychiatry 168, 1255–1265. 10.1176/appi.ajp.2011.11040557, PMID: 21865528 PMC4118766

[ref88] Grant D. A. Schipper L. M. (1952). The acquisition and extinction of conditioned eyelid responses as a function of the percentage of fixed-ratio random reinforcement. J. Exp. Psychol. 43, 313–320. 10.1037/h0057186, PMID: 14946341

[ref89] Grant D. A. Riopelle A. J. Hake H. W. (1950). Resistance to extinction and the pattern of reinforcement. I. Alternation of reinforcement and the conditioned eyelid response. J. Exp. Psychol. 40, 53–60. 10.1037/h0061050 14841332

[ref90] Greer T. L. Trivedi M. H. Thompson L. T. (2005). Impaired delay and trace eyeblink conditioning performance in major depressive disorder. J. Affect Disord. 86, 235–245. 10.1016/j.jad.2005.02.006, PMID: 15935243

[ref91] Grillon C. Morgan C. A. III. (1999). Fear-potentiated startle conditioning to explicit and contextual cues in Gulf War veterans with posttraumatic stress disorder. J. Abnorm. Psychol. 108, 134. 10.1037/0021-843X.108.1.134 10066999

[ref92] Grillon C. Merikangas K. R. Dierker L. Snidman N. Arriaga R. I. Kagan J.. (1999). Startle potentiation by threat of aversive stimuli and darkness in adolescents: a multi-site study. Int. J. Psychophysiol. 32, 63–73. 10.1016/S0167-8760(99)00002-1, PMID: 10192009

[ref93] Grupe D. W. Nitschke J. B. (2013). Uncertainty and anticipation in anxiety: an integrated neurobiological and psychological perspective. Nat. Rev. Neurosci. 14, 488–501. 10.1038/nrn3524, PMID: 23783199 PMC4276319

[ref94] Guillaumin S. Dahhaoui M. Caston J. (1991). Cerebellum and memory: an experimental study in the rat using a passive avoidance conditioning test. Physiol. Behav. 49, 507–511. 10.1016/0031-9384(91)90272-P, PMID: 2062926

[ref95] Guthrie R. M. Bryant R. A. (2006). Extinction learning before trauma and subsequent posttraumatic stress. Psychosom. Med. 68, 307–311. 10.1097/01.psy.0000208629.67653.cc, PMID: 16554398

[ref96] Hake H. W. Grant D. A. (1951). Resistance to extinction and the pattern of reinforcement: II. Effect of successive alternation of blocks of reinforced and unreinforced trials upon the conditioned eyelid response to light. J. Exp. Psychol. 41, 216–220. 10.1037/h0061050, PMID: 14841332

[ref97] Handy J. D. Avcu P. Ko N. Ortiz A. Doria M. J. Servatius R. J. (2018). Facilitated acquisition of the classically conditioned eyeblink response in active duty military expressing posttraumatic stress disorder symptoms. Behav. Brain Res. 339, 106–113. 10.1016/j.bbr.2017.11.014 29154809

[ref98] Hartman T. F. Grant D. A. (1960). Effect of intermittent reinforcement on acquisition, extinction, and spontaneous recovery of the conditioned eyelid response. J. Exp. Psychol. 60, 89–96. 10.1037/h0039832, PMID: 14400148

[ref99] Hineline P. N. (1978a). Warmup in avoidance as a function of time since prior training. J. Exp. Anal. Behav. 29, 87–103. 16812042 10.1901/jeab.1978.29-87 PMC1332811

[ref100] Hineline P. N. (1978b). Warm up in free-operant avoidance as a function of the response-shock shock-shock interval. J. Exp. Anal. Behav. 30, 281–291. 16812108 10.1901/jeab.1978.30-281 PMC1332772

[ref101] Hirshfeld D. R. Rosenbaum J. F. Biederman J. Bolduc E. A. Faraone S. V. Snidman N. (1992). Stable behavioral inhibition and its association with anxiety disorder. J. Am. Acad. Child Adolesc. Psychiatry 31, 103–111. 1537760 10.1097/00004583-199201000-00016

[ref102] Holloway J. L. Trivedi P. Myers C. E. Servatius R. J. (2012). Enhanced conditioned eyeblink response acquisition and proactive interference in anxiety vulnerable individuals. Front. Behav. Neurosci. 6:76. 10.3389/fnbeh.2012.00076, PMID: 23162449 PMC3499707

[ref103] Holloway J. Allen M. T. Myers C. E. Servatius R. J. (2014). Behaviorally inhibited individuals demonstrate significantly enhanced conditioned response acquisition under non-optimal learning conditions. Behav. Brain Res. 261, 49–55. 10.1016/j.bbr.2013.10.041, PMID: 24275381

[ref104] Humphreys L. G. (1939). The effect of random alternation of reinforcement on the acquisition and extinction of conditioned eyelid reactions. J. Exp. Psychol. 25, 141–158. 10.1037/h0058138

[ref105] Jacobson I. G. Donoho C. J. Crum-Cianflone N. F. Maguen S. (2015). Longitudinal assessment of gender differences in the development of PTSD among US military personnel deployed in support of the operations in Iraq and Afghanistan. J. Psychiatr. Res. 68, 30–36. 10.1016/j.jpsychires.2015.05.015, PMID: 26228397

[ref106] Janke K. L. Cominski T. P. Kuzhikandathil E. V. Servatius R. J. Pang K. C. (2015). Investigating the role of hippocampal BDNF in anxiety vulnerability using classical eyeblink c onditioning. Front. Psychiatry 6:106. 10.3389/fpsyt.2015.00106, PMID: 26257661 PMC4513557

[ref107] Jiao X. Beck K. D. Pang K. C. H. Servatius R. J. (2011a). “Animal models of anxiety vulnerability—the Wistar Kyoto rat” in Different views of anxiety disorders. London: Intech. 10.5772/18462

[ref108] Jiao X. Pang K. C. Beck K. D. Minor T. R. Servatius R. J. (2011b). Avoidance perseveration during extinction training in Wistar-Kyoto rats: an interaction of innate vulnerability and stressor intensity. Behav. Brain Res. 221, 98–107. 21376086 10.1016/j.bbr.2011.02.029 PMC3079807

[ref109] Jiao X. Beck K. D. Stewart A. L. Smith I. M. Myers C. E. Servatius R. J.. (2014). Effects of psychotropic agents on extinction of lever-press avoidance in a rat model of anxiety vulnerability. Front. Behav. Neurosci. 8:322. 10.3389/fnbeh.2014.00322, PMID: 25309372 PMC4163983

[ref110] Joseph J. S. Gray M. J. (2008). Exposure therapy for posttraumatic stress disorder. J. Behav. Anal. Offender Vict. Treat. Prevent. 1, 69–79. 10.1037/h0100457

[ref111] Jovanovic T. Norrholm S. D. Blanding N. Q. Davis M. Duncan E. Bradley B.. (2010). Impaired fear inhibition is a biomarker of PTSD but not depression. Depress. Anxiety 27, 244–251. 10.1002/da.20663, PMID: 20143428 PMC2841213

[ref112] Kagan J. Reznick J. S. Snidman N. (1987). The physiology and psychology of behavioral inhibition in children. Child Dev. 58, 1459–1473. 10.2307/1130685, PMID: 3691195

[ref113] Kalin N. H. Shelton S. E. (2003). Nonhuman primate models to study anxiety, emotion regulation, and psychopathology. Ann. N.Y. Acad. Sci. 1008, 189–200. 10.1196/annals.1301.021 14998885

[ref114] Kalin N. H. Shelton S. E. Davidson R. J. (2000). Cerebrospinal fluid corticotropinreleasing hormone levels are elevated in monkeys with patterns of brain activity associated with fearful temperament. Biol. Psychiatry 47, 579–585. 10.1016/S0006-3223(99)00256-5, PMID: 10745049

[ref115] Karamustafalioglu O. K. Zohar J. Güveli M. Gal G. Bakim B. Fostick L.. (2006). Natural course of posttraumatic stress disorder: a 20-month prospective study of Turkish earthquake survivors. J. Clin. Psychol. 67, 882–889. 10.4088/JCP.v67n0604, PMID: 16848647

[ref116] Kashdan T. B. Elhai J. D. Frueh B. C. (2006). Anhedonia and emotional numbing in combat veterans with PTSD. Behav. Res. Ther. 44, 457–467. 10.1016/j.brat.2005.03.001, PMID: 16446151

[ref117] Kashdan T. B. Morina N. Priebe S. (2009). Post-traumatic stress disorder, social anxiety disorder, and depression in survivors of the Kosovo War: experiential avoidance as a contributor to distress and quality of life. J. Anxiety Disord. 23, 185–196. 10.1016/j.janxdis.2008.06.006, PMID: 18676121 PMC2667796

[ref118] Keller M. C. Coventry W. L. Heath A. C. Martin N. G. (2005). Widespread evidence for non-additive genetic variation in Cloninger’s and Eysenck’s personality dimensions using a twin plus sibling design. Behav. Gen. 35, 707. 10.1007/s10519-005-6041-7, PMID: 16273321

[ref119] Keltikangas-Jarvinen L. Kettunen J. Ravaja N. Naatanen P. (1999). Inhibited and disinhibited temperament and autonomic stress reactivity. Int. J. Psychophysiol. 33, 185–196. 10.1016/S0167-8760(99)00057-4, PMID: 10533835

[ref120] Kendler K. S. Myers J. Prescott C. A. (2002). The etiology of phobias: an evaluation of the stress-diathesis model. Arch. Gen. Psychiatry 59, 242–248. 10.1001/archpsyc.59.3.242, PMID: 11879162

[ref121] Kessler R. C. Sonnega A. Bromet E. Hughes M. Nelson C. B. (1995). Posttraumatic stress disorder in the National Comorbidity Survey. Arch. Gen. Psychiatry 52, 1048–1060. 10.1001/archpsyc.1995.03950240066012, PMID: 7492257

[ref123] Kronforst-Collins M. A. Disterhoft J. F. (1998). Lesions of the caudal area of rabbit medial prefrontal cortex impair trace eyeblink conditioning. Neurobiol. Learn. Mem. 69, 147–162. 10.1006/nlme.1997.3818, PMID: 9619994

[ref124] LeDoux J. E. Moscarello J. Sears R. Campese V. (2017). The birth, death and resurrection of avoidance: a reconceptualization of a troubled paradigm. Mol. Psychiatry 22, 24–36. 10.1038/mp.2016.166, PMID: 27752080 PMC5173426

[ref125] Lemos J. C. Zhang G. Walsh T. Kirby L. G. Akanwa A. Brooks-Kayal A.. (2011). Stress-hyperresponsive WKY rats demonstrate depressed dorsal raphe neuronal excitability and dysregulated CRF-mediated responses. Neuropsychopharmacology 36, 721–734. 10.1038/npp.2010.200, PMID: 21160465 PMC3055727

[ref126] Leonard D. W. (1975). Partial reinforcement effects in classical aversive conditioning in rabbits and human beings. J. Comp. Physiol. Psychol. 88, 596–608. 10.1037/h0076419, PMID: 1150940

[ref127] Leonard D. W. Theios J. (1967). Classical eyelid conditioning in rabbits under prolonged single alternation conditions of reinforcement. J. Comp. Physiol. Psychol. 64, 273–276. 10.1037/h0088043, PMID: 6050574

[ref128] Lewis A. H. Niznikiewicz M. A. Delamater A. R. Delgado M. R. (2013). Avoidance-based human Pavlovian-to-instrumental transfer. Eur. J. Neurosci. 38, 3740–3748. 10.1111/ejn.12377, PMID: 24118624 PMC3865081

[ref129] Liberzon I. Sripada C. S. (2008). The functional neuroanatomy of PTSD: a critical review. Prog. Brain Res. 167, 151–169. 10.1016/S0079-6123(07)67011-3, PMID: 18037013

[ref130] Lommen M. J. J. Engelhard I. M. Sijbrandij M. van den Hout M. A. Hermans D. (2013). Pre-trauma individual differences in extinction learning predict posttraumatic stress. Behav. Res. Ther. 51, 63–67. 10.1016/j.brat.2012.11.004, PMID: 23261706

[ref131] Longenecker E. D. Krauskopf J. Bitterman M. E. (1952). Extinction following alternating and random partial reinforcement. Am. J. Psychol. 65, 580–587. 10.2307/1418038, PMID: 12996695

[ref132] Lonigan C. J. Shannon M. P. Taylor C. M. Finch A. J. Sallee F. R. (1994). Children exposed to disaster: II. Risk factors for the development of post-traumatic symptomatology. J. Am. Acad. Child Adolesc. Psychiatry 33, 94–105. 8138526 10.1097/00004583-199401000-00013

[ref777] Lovibond P. (2006). “Fear and Avoidance: An Integrated Expectancy Model” in Fear and learning: From basic processes to clinical implications. eds. Craske M. G. Hermans D. Vansteenwegen D. (Washington, DC, US: American Psychological Association), 117–132. 10.1037/11474-006

[ref133] Lovibond P. F. Saunders J. C. Weidemann G. Mitchell C. J. (2008). Evidence for expectancy as a mediator of avoidance and anxiety in a laboratory model of human avoidance learning. Q. J. Exp. Psychol. 61, 1199–1216. 10.1080/17470210701503229 18938780

[ref134] Lovibond P. F. Mitchell C. J. Minard E. Brady A. Menzies R. G. (2009). Safety behaviours preserve threat beliefs: protection from extinction of human fear conditioning by an avoidance response. Behav. Res. Ther. 47, 716–720. 10.1016/j.brat.2009.04.013, PMID: 19457472

[ref135] Lovibond P. F. Chen S. X. Mitchell C. J. Weidemann G. (2013). Competition betweenan avoidance response and a safety signal: evidence for a single learning system. Biol. Psychol. 92, 9–16. 10.1016/j.biopsycho.2011.09.007, PMID: 21964284

[ref136] Ludewig S. Ludewig K. Geyer M. A. Hell D. Vollenweider F. X. (2002). Prepulse inhibition deficits in patients with panic disorder. Depress. Anxiety 15, 55–60. 10.1002/da.10026, PMID: 11891993

[ref137] Maes M. Delmeire L. Schotte C. Janca A. Creten T. Mylle J.. (1998). The two-factorial symptom structure of post-traumatic stress disorder: depression–avoidance and arousal–anxiety. Psychiatry Res. 81, 195–210. 10.1016/S0165-1781(98)00094-8, PMID: 9858036

[ref138] Marmar C. R. (1996). Characteristics of emergency services personnel related to peritraumatic dissociation during critical incident exposure. Am. J. Psychiatry 153, 94–102. 10.1176/ajp.153.7.94, PMID: 8659646

[ref139] Marshall R. D. Turner J. B. Lewis-Fernandez R. Koenan K. Neria Y. Dohrenwend B. P. (2006). Symptom patterns associated with chronic PTSD in male veterans: new findings from the National Vietnam Veterans Readjustment Study. J. Nerv. Ment. Dis. 194, 275–278. 10.1097/01.nmd.0000207363.25750.56, PMID: 16614549

[ref140] Mathews A. (1994). Cognitive approaches to emotion and emotional disorders. Annu. Rev. Psychol. 45, 25–50. 10.1146/annurev.ps.45.020194.000325, PMID: 8135504

[ref141] Mauk M. D. Thompson R. F. (1987). Retention of classically conditioned eyelid responses following acute decerebration. Brain Res. 403, 89–95. 10.1016/0006-8993(87)90126-0, PMID: 3828818

[ref142] McAuley J. D. Stewart A. L. Webber E. S. Cromwell H. C. Servatius R. J. Pang K. C. H. (2009). Wistar-kyoto rats as an animal model of anxiety vulnerability: support for a hypervigilance hypothesis. Behav. Brain Res. 204, 162–168. 10.1016/j.bbr.2009.05.036, PMID: 19523988 PMC2723189

[ref143] McGlinchey-Berroth R. Carrillo M. C. Gabrieli J. D. Brawn C. M. Disterhoft J. F. (1997). Impaired trace eyeblink conditioning in bilateral, medial-temporal lobe amnesia. Behav. Neurosci. 111, 873–882. 10.1037/0735-7044.111.5.873, PMID: 9383510

[ref144] McNally R. J. (1996). “Cognitive bias in the anxiety disorders” in Perspectives on anxiety, panic, and fear. eds. Hope D. A. Izard C. E. McNally R. J. (Lincoln, NE: University of Nebraska Press), 211–250. 8912310

[ref145] Mineka S. (2004). “The positive and negative consequences of worry in the aetiology of generalized anxiety disorder: a learning theory perspective” in Cognition, emotion and psychopathology theoretical, empirical and clinical directions. (Cambridge, UK: Cambridge University Press), 29–48.

[ref146] Mineka S. Zinbarg R. (2006). A contemporary learning theory perspective on the etiology of anxiety disorders: it’s not what you thought it was. Am. Psychol. 61, 10–26. 10.1037/0003-066X.61.1.10, PMID: 16435973

[ref147] Mobbs D. Petrovic P. Marchant J. L. Hassabis D. Weiskopf N. Seymour B.. (2007). When fear is near: threat imminence elicits prefrontal-periaqueductal gray shifts in humans. Science 317, 1079–1083. 10.1126/science.1144298, PMID: 17717184 PMC2648508

[ref148] Molet M. Leconte C. Rosas J. M. (2006). Acquisition, extinction and temporal discrimination in human conditioned avoidance. Behav. Proc. 73, 199–208. 10.1016/j.beproc.2006.05.009, PMID: 16806735

[ref149] Mommersteeg P. M. Denollet J. Kavelaars A. Geuze E. Vermetten E. Heijnen C. J. (2011). Type D personality, temperament, and mental health in military personnel awaiting deployment. Int. J. Behav. Med. 18, 131–138. 10.1007/s12529-010-9096-7, PMID: 20473600 PMC3088830

[ref150] Moore J. W. Gormezano I. (1961). Yoked comparisons of instrumental and classical eyelid conditioning. J. Exp. Psychol. 62, 552–559. 10.1037/h0044551, PMID: 14475688

[ref151] Moore J. W. Gormezano I. (1963). Effects of omitted versus delayed UCS on classical eyelid conditioning under partial reinforcement. J. Exp. Psychol. 65, 248–257. 10.1037/h0040671

[ref152] Moreno-Rius J. (2018). The cerebellum in fear and anxiety-related disorders. Prog. Neuropsychopharmacol. Biol. Psychiatry. 85, 23–32. 10.1080/17470210701503229 29627508

[ref153] Morgan B. E. (2006). Behavioral inhibition: a neurobiological perspective. Curr. Psychiatry Rep. 8, 270–278. 10.1007/s11920-006-0062-7, PMID: 16879790

[ref154] Morgan C. A. III. Grillon C. Southwick S. M. Davis M. Charney D. S. (1996). Exaggerated acoustic startle reflex in Gulf War veterans with posttraumatic stress disorder. Am. J. Psychiatry 153, 64–68. 10.1176/ajp.153.1.64, PMID: 8540594

[ref155] Mosing M. A. Pedersen N. L. Cesarini D. Johannesson M. Magnusson P. K. Nakamura J.. (2012). Genetic and environmental influences on the relationship between flow proneness, locus of control and behavioral inhibition. PLoS One 7:e47958. 10.1371/journal.pone.0047958, PMID: 23133606 PMC3487896

[ref156] Moustafa A. A. Wufong E. Servatius R. J. Pang K. C. H. Gluck M. A. Myers C. E. (2013). Why trace and delay eyeblink conditioning are sometimes (but not always) hippocampal dependent: a computational model. Brain Res. 1493, 48–67. 10.1016/j.brainres.2012.11.020, PMID: 23178699 PMC3873755

[ref157] Moyer J. R. Deyo R. A. Disterhoft J. F. (1990). Hippocampectomy disrupts trace eye-blink conditioning in rabbits. Behav. Neurosci. 104, 243–252. 10.1037/0735-7044.104.2.243, PMID: 2346619

[ref158] Myers C. E. Ermita B. Harris K. Hasselmo M. Solomon P. Gluck M. (1996). A computational model of the effects of septohippocampal disruption on classical eyeblink conditioning. Neurobiol. Learn. Mem. 66, 51–66. 10.1006/nlme.1996.0043, PMID: 8661251

[ref159] Myers C. E. VanMeenen K. McAuley J. D. Beck K. D. Pang K. C. H. Servatius R. J. (2012a). Facilitated acquisition of eyeblink conditioning in veterans with high behavioral inhibition, a risk factor for post-traumatic stress disorder (PTSD). Stress 15, 31–44. 10.3109/10253890.2011.578184 21790343 PMC3364604

[ref160] Myers C. E. VanMeenen K. M. Servatius R. J. (2012b). Behavioral inhibition and PTSD symptoms in veterans. Psychiatry Res. 196, 271–276. 10.1016/j.psychres.2011.11.015 22397911 PMC3361537

[ref161] Newman F. L. (1967). Differential eyelid conditioning as a function of the probability of reinforcement. J. Exp. Psychol. 75, 412–417. 10.1037/h0025050, PMID: 6079859

[ref162] Nixon S. J. Parsons O. A. (1989). Cloninger’s tridimensional theory of personality: construct validity in a sample of college students. Pers. Individ. Differ. 10, 1261–1267. 10.1016/0191-8869(89)90238-9

[ref163] Norrholm S. D. Jovanovic T. (2018). Fear processing, psychophysiology, and PTSD. Harv. Rev. Psychiatry 26, 129–141. 10.1097/HRP.0000000000000189, PMID: 29734227

[ref164] North C. S. Smith E. M. (1990). Post-traumatic stress disorder in disaster survivors. Compr. Ther. 16, 3–9. 2076600

[ref165] North C. S. Nixon S. J. Shariat S. Mallonee S. McMillen J. C. Spitznagel E. L.. (1999). Psychiatric disorders among survivors of the Oklahoma City bombing. J. Am. Med. Assoc. 282, 755–762. 10.1001/jama.282.8.755, PMID: 10463711

[ref166] North C. S. Pfefferbaum B. Tivis L. Kawasaki A. Reddy C. Spitznagel E. L. (2004). The course of post traumatic stress disorder in a follow-up study of survivors of the Oklahoma City bombing. Ann. Clin. Psychiatry 16, 209–215. 10.1080/10401230490522034, PMID: 15702569

[ref167] Nutt D. J. Malizia A. L. (2004). Structural and functional brain changes in posttraumatic stress disorder. J. Clin. Psychiatry 65(Suppl. 1), 11–17. 10.1615/CritRevImmunol.v24.i4.20, PMID: 14728092

[ref168] O’Donnell M. L. Elliott P. Lau W. Creamer M. (2007). PTSD symptom trajectories: from early to chronic response. Behav. Res. Ther. 45, 601–606. 10.1016/j.brat.2006.03.015, PMID: 16712783

[ref169] Ogińska-Bulik N. Langer I. (2007). Osobowość typu D i strategie radzenia sobie ze stresem a nasilenie objawów PTSD w grupie strażaków [Type D personality, coping with stress and intensity of PTSD symptoms in firefighters]. Med. Pr. 58, 307–316. 18041200

[ref170] Oglesby M. E. Boffa J. W. Short N. A. Raines A. M. Schmidt N. B. (2016). Intolerance of uncertainty as a predictor of post-traumatic stress symptoms following a traumatic event. J. Anxiety Disord. 41, 82–87. 10.1016/j.janxdis.2016.01.005, PMID: 26803928

[ref171] Olton D. S. (1973). Shock-motivated avoidance and the analysis of behavior. Psychopharmacol. Bull. 79, 243–251. 10.1037/h0033902, PMID: 4633560

[ref172] Orr S. P. Pitman R. K. Lasko N. B. Herz L. R. (1993). Psychophysiological assessment of posttraumatic stress disorder imagery in World War II and Korean combat veterans. J. Abnorm. Psychol. 102, 152–159. 10.1037/0021-843X.102.1.152, PMID: 8436691

[ref173] Orr S. P. Metzger L. J. Lasko N. B. Macklin M. L. Peri T. Pitman R. K. (2000). De novo conditioning in trauma-exposed individuals with and without posttraumatic stress disorder. J. Abnorm. Psychol. 109, 290–298. 10.1037/0021-843X.109.2.290, PMID: 10895567

[ref174] Orr S. P. Milad M. R. Metzger L. J. Lasko N. B. Gilbertson M. W. Pitman R. K. (2006). Effects of beta blockade, PTSD diagnosis, and explicit threat on the extinction and retention of an aversively conditioned response. Biol. Psychol. 73, 262–271. 10.1016/j.biopsycho.2006.05.001, PMID: 16828533

[ref175] Orr S. P. Lasko N. B. Macklin M. L. Pineles S. L. Chang Y. Pitman R. K. (2012). Predicting post-trauma stress symptoms from pre-trauma psychophysiologic reactivity, personality traits and measures of psychopathology. Biol. Mood Anxiety Disord. 2. 10.1186/2045-5380-2-8 PMC3412748 22738068

[ref176] Orsillo S. M. Heimberg R. G. Juster H. R. Garrett J. (1996). Social phobia and PTSD in Vietnam veterans. J. Trauma. Stress 9, 235–252. 10.1002/jts.2490090207 8731545

[ref177] Osuch E. A. Benson B. Geraci M. Podell D. Herscovitch P. McCann U. D.. (2001). Regional cerebral blood flow correlated with flashback intensity in patients with posttraumatic stress disorder. Biol. Psychiatry 50, 246–253. 10.1016/S0006-3223(01)01107-6, PMID: 11522258

[ref178] Pai A. Suris A. M. North C. S. (2017). Posttraumatic stress disorder in the DSM-5: controversy, change, and conceptual considerations. Behav. Sci. 7:7. 10.3390/bs7010007, PMID: 28208816 PMC5371751

[ref179] Pantazatos S. P. Talati A. Pavlidis P. Hirsch J. (2012). Cortical functional connectivity decodes subconscious, task-irrelevant threat-related emotion processing. Neuroimage 61, 1355–1363. 10.1016/j.neuroimage.2012.03.051, PMID: 22484206 PMC3393600

[ref180] Paré W. P. (1989). “Behavioral despair” test predicts stress ulcer in WKY rats. Physiol. Behav. 46, 483–487. 10.1016/0031-9384(89)90025-5, PMID: 2623074

[ref181] Paré W. P. (1992). The performance of WKY rats on three tests of emotional behavior. Physiol. Behav. 51, 1051–1056. 10.1016/0031-9384(92)90091-F, PMID: 1615043

[ref182] Paré W. P. (1993). Passive-avoidance behavior in Wistar-Kyoto (WKY), Wistar, and Fischer-344 rats. Physiol. Behav. 54, 845–852. 10.1016/0031-9384(93)90291-M, PMID: 8248372

[ref183] Paré W. P. (1994). Open field, learned helplessness, conditioned defensive burying, and forced swim tests in wky rats. Physiol. Behav. 55, 433–439. 10.1016/0031-9384(94)90097-3, PMID: 8190758

[ref184] Paré W. P. (1996). Enhanced retrieval of unpleasant memories influenced by shock controllability, shock sequence, and rat strain. Biol. Psychiatry 39, 808–813. 10.1016/0006-3223(95)00220-0, PMID: 8731522

[ref185] Pedersen S. S. van Domburg R. T. Theuns D. A. Jordaens L. Erdman R. A. (2004). Type D personality is associated with increased anxiety and depressive symptoms in patients with an implantable cardioverter defibrillator and their partners. Psychosom. Med. 66, 714–719. 10.1097/01.psy.0000132874.52202.21, PMID: 15385696

[ref186] Perez-Edgar K. Roberson-Nay R. Hardin M. G. Poeth K. Guyer A. E. Nelson E. E.. (2007). Attention alters neural responses to evocative faces in behaviorally inhibited adolescents. Neuroimage 35, 1538–1546. 10.1016/j.neuroimage.2007.02.006, PMID: 17376704 PMC2062494

[ref187] Pérez-Edgar K. Bar-Haim Y. McDermott J. M. Chronis-Tuscano A. Pine D. S. Fox N. A. (2010). Attention biases to threat and behavioral inhibition in early childhood shape adolescent social withdrawal. Emotion 10, 349–357. 10.1037/a0018486, PMID: 20515224 PMC3614079

[ref188] Peri T. Ben-Shakhar G. Orr S. P. Shalev A. Y. (2000). Psychophysiologic assessment of aversive conditioning in posttraumatic stress disorder. Biol. Psychiatry 47, 512–519. 10.1016/S0006-3223(99)00144-4, PMID: 10715357

[ref189] Perrotti L. I. Dennis T. S. Jiao X. Servatius R. J. Pang K. C. Beck K. D. (2013). Activation of extracellular signal-regulated kinase (ERK) and ΔFosB in emotion-associated neural circuitry after asymptotic levels of active avoidance behavior are attained. Brain Res. Bull. 98, 102–110. 10.1016/j.brainresbull.2013.07.004, PMID: 23932962

[ref190] Perry S. L. Moore J. W. (1965). The partial-reinforcement effect sustained through blocks of continuous reinforcement in classical eyelid conditioning. J. Exp. Psychol. 69, 158. 10.1037/h0021587, PMID: 14279772

[ref191] Phipps S. Jurbergs N. Long A. (2009). Symptoms of post-traumatic stress in children with cancer: does personality trump health status? Psycho-Oncology 18, 992–1002. 10.1002/pon.1496, PMID: 19177432 PMC2735607

[ref192] Pineles S. L. Blumenthal T. D. Curreri A. J. Nillni Y. I. Putnam K. M. Resick P. A.. (2016). Prepulse inhibition deficits in women with PTSD. Psychophysiology 53, 1377–1385. 10.1111/psyp.12679, PMID: 27237725

[ref193] Pitman R. K. (1988). Post-traumatic stress disorder, conditioning, and network theory. Psychiatr. Ann. 18, 182–189. 10.3928/0048-5713-19880301-11

[ref194] Pitman R. K. Orr S. P. (1986). Test of the conditioning model of neurosis: differential aversive conditioning of angry and neutral facial expressions in anxiety disorder patients. J. Abnorm. Psychol. 95, 208–213. 10.1037/0021-843X.95.3.208, PMID: 3745641

[ref195] Pitman R. K. Orr S. P. Forgue D. F. Altman B. de Jong J. B. Herz L. R. (1987). Psychophysiologic assessment of posttraumatic stress disorder imagery in Vietnam combat veterans. Arch. Gen. Psychiatry 44, 970–975. 10.1001/archpsyc.1987.01800230050009, PMID: 3675137

[ref196] Pitman R. K. Gilbertson M. W. Gurvits T. V. May F. S. Lasko N. B. Metzger L. J. (2006). Clarifying the origin of biological abnormalities in PTSD through the study of identical twins discordant for combat exposure. Ann. N.Y. Acad. Sci. 1071, 242–254. 10.1196/annals.1364.019 16891575 PMC2770249

[ref197] Pittig A. Treanor M. LeBeau R. T. Craske M. G. (2018). The role of associative fear and avoidance learning in anxiety disorders: gaps and directions for future research. Neurosci. Biobehav. Rev. 88, 117–140. 10.1016/j.neubiorev.2018.03.015, PMID: 29550209

[ref198] Port R. L. Mikhail A. A. Patterson M. M. (1985). Differential effects of hippocampectomy on classically conditioned rabbit nictitating membrane response related to interstimulus interval. Behav. Neurosci. 99, 200–208. 10.1037/0735-7044.99.2.200, PMID: 3843707

[ref199] Price L. E. Abbott D. W. Vandament W. E. (1965). Effects of CS and UCS change on extinction of the conditioned eyelid response. J. Exp. Psychol. 69, 437–438. 10.1037/h0021769, PMID: 14286317

[ref200] Prindaville P. Stein N. (1978). Predictability, controllability, and inoculation against learned helplessness. Behav. Res. Ther. 16, 263–271. 10.1016/0005-7967(78)90024-4

[ref201] Prut L. Belzung C. (2003). The open field as a paradigm to measure the effects of drugs on anxiety-like behaviors: a review. Eur. J. Pharmacol. 463, 3–33. 10.1016/S0014-2999(03)01272-X, PMID: 12600700

[ref202] Qouta S. Punamäki R. L. Montgomery E. El Sarraj E. (2007). Predictors of psychological distress and positive resources among Palestinian adolescents: trauma, child, and mothering characteristics. Child Abuse Negl. 3, 699–717. 10.1016/j.chiabu.2005.07.007 17628671

[ref203] Rabellino D. Densmore M. Théberge J. McKinnon M. C. Lanius R. A. (2018). The cerebellum after trauma: resting-state functional connectivity of the cerebellum in posttraumatic stress disorder and its dissociative subtype. Hum. Brain Mapp. 39, 3354–3374. 10.1002/hbm.24081, PMID: 29667267 PMC6866303

[ref204] Radell M. L. Sanchez R. Weinflash N. Myers C. E. (2016). The personality trait of behavioral inhibition modulates perceptions of moral character and performance during the trust game: behavioral results and computational modeling. PeerJ 4:e1631. 10.7717/peerj.1631, PMID: 27004148 PMC4800786

[ref205] Rademaker A. R. van Zuiden M. Vermetten E. Geuze E. (2011). Type D personality and the development of PTSD symptoms: a prospective study. J. Abnorm. Psychol. 120, 299–307. 10.1037/a0021806 21171726

[ref206] Reynolds W. F. (1958). Acquisition and extinction of the conditioned eyelid response following partial and continuous reinforcement. J. Exp. Psychol. 55, 335–341. 10.1037/h0042202, PMID: 13539314

[ref207] Ricart T. M. De Niear M. A. Jiao X. Pang K. C. Beck K. D. Servatius R. J. (2011a). Deficient proactive interference of eyeblink conditioning in Wistar-Kyoto rats. Behav. Brain Res. 216, 59–65. 10.1016/j.bbr.2010.07.005 20621128 PMC2975831

[ref208] Ricart T. M. Jiao X. Pang K. C. Beck K. D. Servatius R. J. (2011b). Classical and instrumental conditioning of eyeblink responses in Wistar–Kyoto and Sprague–Dawley rats. Behav. Brain Res. 216, 414–418. 10.1016/j.bbr.2010.08.029 20801161 PMC3057661

[ref209] Ricart T. M. Servatius R. J. Beck K. D. (2012). “Acquisition of active avoidance behavior as a precursor to changes in general arousal in an animal model of PTSD” in Post traumatic stress disorders in a global context. London: InTech.

[ref210] Ristvedt S. L. Trinkaus K. M. (2009). Trait anxiety as an independent predictor of poor health-related quality of life and post-traumatic stress symptoms in rectal cancer. Br. J. Health Psychol. 14, 701–715. 10.1348/135910708X400462, PMID: 19171084 PMC2756319

[ref211] Roland J. J. Janke K. L. Servatius R. J. Pang K. C. (2014). GABAergic neurons in the medial septum-diagonal band of Broca (MSDB) are important for acquisition of the classically conditioned eyeblink response. Brain Struct. Funct. 219, 1231–1237. 10.1007/s00429-013-0560-4, PMID: 24965560 PMC4073235

[ref212] Ross L. E. (1959). The decremental effects of partial reinforcement during acquisition of the conditioned eyelid response. J. Exp. Psychol. 57, 74–82. 10.1037/h0046965, PMID: 13641576

[ref213] Ross L. E. Spence K. W. (1960). Eyelid conditioning performance under partial reinforcement as a function of UCS intensity. J. Exp. Psychol. 59, 379–382. 10.1037/h0043717, PMID: 14439169

[ref214] Rubin D. C. Berntsen D. Bohni M. K. (2008). A memory-based model of posttraumatic stress disorder: evaluating basic assumptions underlying the PTSD diagnosis. Psychol. Rev. 115, 985–1011. 10.1037/a0013397 18954211 PMC2762652

[ref215] Runquist W. N. (1963). Performance in eyelid conditioning following changes in reinforcement schedule. J. Exp. Psychol. 65, 617–618. 10.1037/h0048016, PMID: 13975554

[ref216] Samanez-Larkin G. R. Hollon N. G. Carstensen L. L. Knutson B. (2008). Individual differences in insular sensitivity during loss anticipation predict avoidance learning. Psychol. Sci. 19, 320–323. 10.1111/j.1467-9280.2008.02087.x, PMID: 18399882 PMC2365707

[ref217] Schaefer K. A. Malta L. S. Dörfel D. Rohleder N. Werner A. (2006). A meta-analysis of structural brain abnormalities in PTSD. Neurosci. Biobehav. Rev. 30, 1004–1031. 10.1016/j.neubiorev.2006.03.004 16730374

[ref218] Schlund M. W. Magee S. Hudgins C. D. (2011). Human avoidance and approach learning: evidence for overlapping neural systems and experiential avoidance modulation of avoidance neurocircuitry. Behav. Brain Res. 225, 437–448. 10.1016/j.bbr.2011.07.054, PMID: 21840340

[ref219] Schmaltz L. W. Giulian D. (1972). Faster acquisition of discriminated lever-press avoidance by hippocampectomized rats. J. Comp. Physiol. Psychol. 81, 149–154. 10.1037/h0033325, PMID: 5074302

[ref220] Schmaltz L. W. Theios J. (1972). Acquisition and extinction of a classically conditioned response in hippocampectomized rabbits (*Oryctolagus cuniculus*). J. Comp. Physiol. Psychol. 79, 328–333. 10.1037/h0032531, PMID: 5026000

[ref221] Schreurs B. G. Burhans L. B. (2015). Eyeblink classical conditioning and post-traumatic stress disorder—a model systems approach. Front. Psychiatry, 6:50. 10.3389/fpsyt.2015.00050, PMID: 25904874 PMC4389289

[ref222] Schutter D. J. (2013). “Human cerebellum in motivation and emotion” in Handbook of the cerebellum and cerebellar disorders. (Netherlands: Springer), 1771–1782.

[ref223] Schwartz C. E. Wright C. I. Shin L. M. Kagan J. Rauch S. L. (2003a). Inhibited and uninhibited infants “grown up” adult amygdalar response to novelty. Science 300, 1952–1953. 10.1126/science.1083703 12817151

[ref224] Schwartz C. E. Wright C. I. Shin L. M. Kagan J. Whalen P. J. McMullin K. G. (2003b). Differential amygdalar response to novel versus newly familiar neutral faces: a functional MRI probe developed for studying inhibited temperament. Biol. Psychiatry 53, 854–862. 10.1016/S0006-3223(02)01906-6 12742672

[ref225] Seng J. S. Low L. M. K. Sperlich M. Ronis D. L. Liberzon I. (2009). Prevalence, trauma history, and risk for posttraumatic stress disorder among nulliparous women in maternity care. Obstet. Gynecol. 114, 839–847. 10.1097/AOG.0b013e3181b8f8a2, PMID: 19888043 PMC3124073

[ref888] Servatius R. J. (2016). Avoidance: from basic science to psychopathology. Front. Behav. Neurosci. 10:15. 10.3389/fnbeh.2016.00015 26903831 PMC4751251

[ref226] Servatius R. J. Ottenweller J. E. Beldowicz D. Guo W. Zhu G. Natelson B. H. (1998). Persistently exaggerated startle responses in rats treated with pyridostigmine bromide. J. Pharmacol. Exp. Ther. 287, 1020–1028. 9864288

[ref227] Servatius R. J. Jiao X. Beck K. D. Pang K. C. H. Minor T. R. (2008). Rapid avoidance acquisition in Wistar–Kyoto rats. Behav. Brain Res. 192, 191–197. 10.1016/j.bbr.2008.04.006, PMID: 18501974

[ref228] Servatius R. J. Avcu P. Ko N. Jiao X. Beck K. D. Minor T. R.. (2015). Avoidance expression in rats as a function of signal-shock interval: strain and sex differences. Front. Behav. Neurosci. 9:168. 10.3389/fnbeh.2015.00168, PMID: 26217200 PMC4491620

[ref229] Servatius R. J. Handy J. D. Doria M. J. Myers C. E. Marx C. E. Lipsky R. (2017). Stress-related mental health symptoms in Coast Guard: incidence, vulnerability, and neurocognitive performance. Front. Psychol. 8:1513. 10.3389/fpsyg.2017.01513 28959220 PMC5603677

[ref230] Shalev A. Y. Galai T. Eth S. (1993). Levels of trauma: a multidimensional approach to the treatment of PTSD. Psychiatry 56, 166–177. 8102486 10.1080/00332747.1993.11024631

[ref231] Sheynin J. Beck K. D. Pang K. C. H. Servatius R. J. Shikari S. Ostovich J. (2014a). Behaviourally inhibited temperament and female sex, two vulnerability factors for anxiety disorders, facilitate conditioned avoidance (also) in humans. Behav. Proc. 103, 228–235. 10.1016/j.beproc.2014.01.003 PMC3972301 24412263

[ref232] Sheynin J. Beck K. D. Servatius R. J. Myers C. E. (2014b). Acquisition and extinction of human avoidance behavior: attenuating effect of safety signals and associations with anxiety vulnerabilities. Front. Behav. Neurosci. 8:323. 10.3389/fnbeh.2014.00323 25309373 PMC4163982

[ref233] Sheynin J. Shind C. Radell M. Ebanks-Williams Y. Gilbertson M. W. Beck K. D.. (2017). Greater avoidance behavior in individuals with posttraumatic stress disorder symptoms. Stress 20, 285–293. 10.1080/10253890.2017.1309523, PMID: 28322068 PMC5490437

[ref234] Simeon D. Yehuda R. Cunill R. Knutelska M. Putnam F. W. Smith L. M. (2007). Factors associated with resilience in healthy adults. Psychoneuroendocrinology 32, 1149–1152. 10.1016/j.psyneuen.2007.08.005, PMID: 17913377

[ref235] Simmons A. Strigo I. Matthews S. C. Paulus M. P. Stein M. B. (2006). Anticipation of aversive visual stimuli is associated with increased insula activation in anxiety-prone subjects. Biol. Psychiatry 60, 402–409. 10.1016/j.biopsych.2006.04.038, PMID: 16919527

[ref236] Smith I. M. Pang K. C. Servatius R. J. Jiao X. Beck K. D. (2016). Paired-housing selectively facilitates within-session extinction of avoidance behavior, and increases c-Fos expression in the medial prefrontal cortex, in anxiety vulnerable Wistar-Kyoto rats. Physiol. Behav. 164, 198–206. 10.1016/j.physbeh.2016.05.044, PMID: 27235339

[ref237] Smith-Bell C. A. Burhans L. B. Schreurs B. G. (2012). Predictors of susceptibility and resilience in an animal model of posttraumatic stress disorder. Behav. Neurosci. 126, 749–761. 10.1037/a0030713, PMID: 23181382 PMC3513376

[ref238] Smoller J. W. Rosenbaum J. F. Biederman J. Kennedy J. Dai D. Racette S. R.. (2003). Association of a genetic marker at the corticotropin-releasing hormone locus with behavioral inhibition. Biol. Psychiatry 54, 1376–1381. 10.1016/S0006-3223(03)00598-5, PMID: 14675801

[ref239] Smoller J. W. Yamaki L. H. Fagerness J. A. Biederman J. Racette S. Laird N. M.. (2005). The corticotropin-releasing hormone gene and behavioral inhibition in children at risk for panic disorder. Biol. Psychiatry 57, 1485–1492. 10.1016/j.biopsych.2005.02.018, PMID: 15953484

[ref240] Solberg L. C. Olson S. L. Turek F. W. Redei E. (2001). Altered hormone levels and circadian rhythm of activity in the WKY rat, a putative animal model of depression. Am. J. Physiol. Regul. Intergr. Comp. Physiol. 281, R786–R794. 10.1152/ajpregu.2001.281.3.R786 11506993

[ref241] Solomon P. Solomon S. Van der Schaaf E. Perry H. (1983). Altered activity in the hippocampus is more detrimental to classical conditioning than removing the structure. Science 220, 329–331. 10.1126/science.6836277, PMID: 6836277

[ref242] Solomon Z. Horesh D. Ein-Dor T. (2009). The longitudinal course of posttraumatic stress disorder symptom clusters among war veterans. J. Clin. Psychiatry 70, 837–843. 10.4088/JCP.08m04347, PMID: 19573481

[ref243] Spence K. W. (1964). Anxiety (drive) level and performance in eyelid conditioning. Psychopharmacol. Bull. 61, 129–139. 10.1037/h0042876, PMID: 14116047

[ref244] Spence K. W. Beecroft R. S. (1954). Differential conditioning and level of anxiety. J. Exp. Psychol. 48, 399–403. 10.1037/h0057825, PMID: 13221734

[ref245] Spence K. W. Farber I. E. (1953). Conditioning and extinction as a function of anxiety. J. Exp. Psychol. 45, 116–119. 10.1037/h0055234, PMID: 13052839

[ref246] Spence K. W. Farber I. E. (1954). The relation of anxiety to differential eyelid conditioning. J. Exp. Psychol. 67, 127–134. 10.1037/h0062162 13143172

[ref247] Spence K. W. Spence J. T. (1966). Sex and anxiety differences in eyelid conditioning. Psychopharmacol. Bull. 65, 137–142. 10.1037/h0022982, PMID: 5325164

[ref248] Spence K. W. Taylor J. (1951). Anxiety and strength of the UCS as determiners of the amount of eyelid conditioning. J. Exp. Psychol. 42, 183–188. 10.1037/h0061580, PMID: 14880670

[ref249] Spence K. W. Taylor J. A. (1953). The relation of conditioned response strength to anxiety in normal, neurotic, and psychotic subjects. J. Exp. Psychol. 45, 265–272. 10.1037/h0056392, PMID: 13052861

[ref250] Spence K. W. Weyant R. G. (1960). Conditioning performance of high-and low-anxious Ss in the absence of a warning signal. J. Exp. Psychol. 60, 146–149. 10.1037/h0043356, PMID: 13833234

[ref251] Spiegler K. M. Fortress A. M. Pang K. C. H. (2018). Differential use of danger and safety signals in an animal model of anxiety vulnerability: the behavioral economics of avoidance. Prog. Neuropsychopharmacol. Biol. Psychiatry 82, 195–204. 10.1016/j.pnpbp.2017.11.015 29175308

[ref252] Spielberger C. D. (1983). Manual for the State-Trait Anxiety Inventory. STAI (Form Y), Palo Alto, CA: Consulting Psychologists Press, Inc.

[ref253] Steinmetz J. E. Logue S. F. Miller D. P. (1993). Using signaled barpressing tasks to study the neural substrates of appetitive and aversive learning in rats: behavioral manipulations and cerebellar lesions. Behav. Neurosci. 107, 941–954. 10.1037/0735-7044.107.6.941, PMID: 8136069

[ref254] Straube T. Schmidt S. Weiss T. Mentzel H. J. Miltner W. H. (2009). Dynamic activation of the anterior cingulate cortex during anticipatory anxiety. Neuroimage 44, 975–981. 10.1016/j.neuroimage.2008.10.022, PMID: 19027072

[ref255] Suslow T. Kugel H. Rauch A. V. Dannlowski U. Bauer J. Konrad C.. (2009). Attachment avoidance modulates neural response to masked facial emotion. Hum. Brain Mapp. 30, 3553–3562. 10.1002/hbm.20778, PMID: 19347874 PMC6870570

[ref256] Sussman D. Pang E. W. Jetly R. Dunkley B. T. Taylor M. J. (2016). Neuroanatomical features in soldiers with post-traumatic stress disorder. BMC Neurosci. 17:13. 10.1186/s12868-016-0247-x 27029195 PMC4815085

[ref257] Svihra M. Katzman M. A. (2004). Behavioural inhibition: a predictor of anxiety. Paediatr. Child Health 9, 547–550. 10.1093/pch/9.8.547, PMID: 19680482 PMC2724161

[ref258] Taylor J. A. (1951). The relationship of anxiety to the conditioned eyelid response. J. Exp. Psychol. 41, 81–92. 10.1037/h0059488, PMID: 14824412

[ref259] Taylor J. A. (1953). A personality scale of manifest anxiety. J. Abnorm. Soc. Psychol. 48, 285–290. 10.1037/h0056264, PMID: 13052352

[ref260] Taylor J. A. (1956). Drive theory and manifest anxiety. Psychopharmacol. Bull. 53, 303–320. 10.1037/h0040353, PMID: 13336198

[ref261] Thompson R. F. Steinmetz J. E. (2009). The role of the cerebellum in classical conditioning of discrete behavioral responses. Neuroscience 162, 732–755. 10.1016/j.neuroscience.2009.01.041, PMID: 19409234

[ref262] Tyrka A. R. Mello A. F. Mello M. F. Gagne G. G. Grover K. E. Anderson G. M.. (2006). Temperament and hypothalamic–pituitary–adrenal axis function in healthy adults. Psychoneuroendocrinology 31, 1036–1045. 10.1016/j.psyneuen.2006.06.004, PMID: 16908106 PMC4469475

[ref263] Tyrka A. R. Wier L. M. Price L. H. Rikhye K. Ross N. S. Anderson G. M.. (2008). Cortisol and ACTH responses to the Dex/CRH test: influence of temperament. Horm. Behav. 53, 518–525. 10.1016/j.yhbeh.2007.12.004, PMID: 18294637 PMC2637444

[ref264] Vythilingam M. Lawley M. Collin C. Bonne O. Agarwal R. Hadd K.. (2006). Hydrocortisone impairs hippocampal-dependent trace eyeblink conditioning in post-traumatic stress disorder. Neuropsychopharmacology 31, 182–188. 10.1038/sj.npp.1300843, PMID: 16123770

[ref265] Wang T. Liu J. Zhang J. Zhan W. Li L. Wu M. (2016). Altered resting-state functional activity in posttraumatic stress disorder: a quantitative meta-analysis. Sci. Rep. 6:27131. 10.1038/srep27131 27251865 PMC4890007

[ref266] Weiss C. Bouwmeester H. Power J. M. Disterhoft J. F. (1999). Hippocampal lesions prevent trace eyeblink conditioning in the freely moving rat. Behav. Brain Res. 99, 123–132. 10.1016/S0166-4328(98)00096-5, PMID: 10512579

[ref267] Weisz D. J. Harden D. G. Xiang Z. (1992). Effects of amygdala lesions on reflex facilitation and conditioned response acquisition during nictitating membrane response conditioning in rabbit. Behav. Neurosci. 106, 262–273. 10.1037/0735-7044.106.2.262, PMID: 1317182

[ref268] Werner N. S. Meindl T. Engel R. R. Rosner R. Riedel M. Reiser M.. (2009). Hippocampal function during associative learning in patients with posttraumatic stress disorder. J. Psychiatr. Res. 43, 309–318. 10.1016/j.jpsychires.2008.03.011, PMID: 18490028

[ref269] Wessa M. Flor H. (2007). Failure of extinction of fear responses in posttraumatic stress disorder: evidence from second-order conditioning. Am. J. Psychiatry 164, 1684–1692. 10.1176/appi.ajp.2007.07030525, PMID: 17974933

[ref270] Xia W. Dymond S. Lloyd K. Vervliet B. (2017). Partial reinforcement of avoidance and resistance to extinction in humans. Behav. Res. Ther. 96, 79–89. 10.1016/j.brat.2017.04.002, PMID: 28416167

[ref271] Zinbarg R. E. Barlow D. H. (1996). Structure of anxiety and the anxiety disorders: a hierarchical model. J. Abnorm. Psychol. 105, 181–193. 10.1037/0021-843X.105.2.181 8722999

[ref272] Zuj D. V. Norrholm S. D. (2019). The clinical applications and practical relevance of human conditioning paradigms for posttraumatic stress disorder. Prog. Neuropsychopharmacol. Biol. Psychiatry 88, 339–351. 10.1016/j.pnpbp.2018.08.014 30134147

[ref273] Zuj D. V. Palmer M. A. Lommen M. J. J. Felmingham K. L. (2016). The centrality of fear extinction in linking risk factors to PTSD: a narrative review. Neurosci. Biobehav. Rev. 69, 15–35. 10.1016/j.neubiorev.2016.07.014, PMID: 27461912

